# Supplementary material for: A cost-effective sequencing method for genetic studies combining high-depth whole exome and low-depth whole genome
Source: NPJ Genom Med. 2024 Feb 7;9:8. doi: 10.1038/s41525-024-00390-3 (PMC10850497; doi:10.1038/s41525-024-00390-3)
Supplement: Supplementary file 1 — Reporting summary [file 41525_2024_390_MOESM1_ESM.pdf]

nature portfolio

Corresponding author(s): Dr Daniel Taliun

Last updated by author(s): Oct 27, 2023

Reporting Summary

Nature Portfolio wishes to improve the reproducibility of the work that we publish. This form provides structure for consistency and transparency in reporting. For further information on Nature Portfolio policies, see our Editorial Policies and the Editorial Policy Checklist.

Statistics

For all statistical analyses, confirm that the following items are present in the figure legend, table legend, main text, or Methods section.

n/a	Confirmed	
☐	☒	The exact sample size (*n*) for each experimental group/condition, given as a discrete number and unit of measurement
☐	☒	A statement on whether measurements were taken from distinct samples or whether the same sample was measured repeatedly
☐	☒	The statistical test(s) used AND whether they are one- or two-sided<br>*Only common tests should be described solely by name; describe more complex techniques in the Methods section.*
☐	☒	A description of all covariates tested
☐	☒	A description of any assumptions or corrections, such as tests of normality and adjustment for multiple comparisons
☐	☒	A full description of the statistical parameters including central tendency (e.g. means) or other basic estimates (e.g. regression coefficient) AND variation (e.g. standard deviation) or associated estimates of uncertainty (e.g. confidence intervals)
☐	☒	For null hypothesis testing, the test statistic (e.g. *F*, *t*, *r*) with confidence intervals, effect sizes, degrees of freedom and *P* value noted<br>*Give P values as exact values whenever suitable.*
☒	☐	For Bayesian analysis, information on the choice of priors and Markov chain Monte Carlo settings
☒	☐	For hierarchical and complex designs, identification of the appropriate level for tests and full reporting of outcomes
☐	☒	Estimates of effect sizes (e.g. Cohen's *d*, Pearson's *r*), indicating how they were calculated

Our web collection on statistics for biologists contains articles on many of the points above.

Software and code

Policy information about availability of computer code

Data collection	Data collection is described in the methods
Data analysis	Tools and software used for genome data processing and analysis are listed and referenced in the Methods. Furthermore the manuscript includes a data & code availability sections listing the code and pipelines used for the analysis, and the URL to access it on github public repositories.

For manuscripts utilizing custom algorithms or software that are central to the research but not yet described in published literature, software must be made available to editors and reviewers. We strongly encourage code deposition in a community repository (e.g. GitHub). See the Nature Portfolio guidelines for submitting code & software for further information.

Data

Policy information about availability of data

All manuscripts must include a data availability statement. This statement should provide the following information, where applicable:

- Accession codes, unique identifiers, or web links for publicly available datasets
- A description of any restrictions on data availability
- For clinical datasets or third party data, please ensure that the statement adheres to our policy

The generated WES, WGS, and WEGS sequencing data for GIAB samples are available for download at https://datahub-778-pbbb.p.genap.ca/. The variant calling and analysis pipelines and source code are publicly available through version control repositories listed in the table below.

Research involving human participants, their data, or biological material

Policy information about studies with human participants or human data. See also policy information about sex, gender (identity/presentation), and sexual orientation and race, ethnicity and racism.

Reporting on sex and gender	Sex is considered as a important biological variable in this study. For WEGS sequencing data processing, we treat the autosomes and sex chromosomes separately, following GATK's best practices. This is described in the Methods.
Reporting on race, ethnicity, or other socially relevant groupings	In this study, we report the self-reported ancestry of the one family trio that we study in depth from the Genome-in-a-bottle (GIAB) consortium. For our validation cohort, we perform genetic ancestry inference analysis, and report the global genetic ancestry groupings.
Population characteristics	See above.
Recruitment	The GIAB trio was recruted and consented by the Personal Genome Project. The hospital-based recruitment of our validation cohort is described in the Methods. Hospital-based cohort are known to display their own biases (more severely impaired patients) relative to population-based cohort, but this should not impact our conclusion.
Ethics oversight	McGill University, Insel University Hospital of Bern, Switzerland

Note that full information on the approval of the study protocol must also be provided in the manuscript.

Field-specific reporting

Please select the one below that is the best fit for your research. If you are not sure, read the appropriate sections before making your selection.

☒ Life sciences ☐ Behavioural & social sciences ☐ Ecological, evolutionary & environmental sciences

For a reference copy of the document with all sections, see nature.com/documents/nr-reporting-summary-flat.pdf

Life sciences study design

All studies must disclose on these points even when the disclosure is negative.

Sample size	The sample size of the benchmark experiment (37 samples sequenced in total) was chosen to be able to have multiple samples for each of the 3 experiments. Sample size of the validation cohort (the PAD) was chosen to maximise the nb of patients studied.
Data exclusions	NA
Replication	The benchmark experiment was specifically designed to have multiple replicates of each experiment.
Randomization	We used randomization for distributing the validation cohort samples into batches.
Blinding	Blinding is not relevant to our study.

Reporting for specific materials, systems and methods

We require information from authors about some types of materials, experimental systems and methods used in many studies. Here, indicate whether each material, system or method listed is relevant to your study. If you are not sure if a list item applies to your research, read the appropriate section before selecting a response.

Materials & experimental systems

n/a	Involved in the study	
☒	☐	Antibodies
☒	☐	Eukaryotic cell lines
☒	☐	Palaeontology and archaeology
☒	☐	Animals and other organisms
☒	☐	Clinical data
☒	☐	Dual use research of concern
☒	☐	Plants

Methods

n/a	Involved in the study	
☒	☐	ChIP-seq
☒	☐	Flow cytometry
☒	☐	MRI-based neuroimaging
