# Supplementary material for: A cost-effective sequencing method for genetic studies combining high-depth whole exome and low-depth whole genome
Source: NPJ Genom Med. 2024 Feb 7;9:8. doi: 10.1038/s41525-024-00390-3 (PMC10850497; doi:10.1038/s41525-024-00390-3)
Supplement: Supplementary file 2 — Supplementary Information [file 41525_2024_390_MOESM2_ESM.pdf]

Supplementary Information

A cost-effective sequencing method for genetic studies combining high-depth whole exome and low-depth whole genome

Claude Bhérer[1,2,3], Robert Eveleigh[2,4], Katerina Trajanoska[1,2,3], Janick St-Cyr[2], Antoine Paccard[2], Praveen Nadukkalam Ravindran[2,3], Elizabeth Caron[2], Nimara Bader Asbah[1,2], Peyton McClelland[1,2,3], Clare Wei[2,3], Iris Baumgartner[5,6], Marc Schindewolf[5,6], Yvonne Döring[5,6,7], Danielle Perley[2,4], François Lefebvre[2,4], Pierre Lepage[2], Mathieu Bourgey[8], Guillaume Bourque[1,2,4], Jiannis Ragoussis[1,2], Vincent Mooser[1,2,3], Daniel Taliun[1,2,3]

[1] Department of Human Genetics, Faculty of Medicine and Health Sciences, McGill University, Montréal, Québec, Canada
[2] Victor Phillip Dahdaleh Institute of Genomic Medicine at McGill University, Montréal, Québec, Canada
[3] Canada Excellence Research Chair in Genomic Medicine, McGill University, Montréal, Québec, Canada
[4] Canadian Centre for Computational Genomics, McGill University, Montréal, Québec, Canada
[5] Division of Angiology, Swiss Cardiovascular Center, Inselspital, Bern University Hospital, University of Bern, Bern, Switzerland
[6] Department for BioMedical Research (DBMR), University of Bern, Switzerland
[7] Institute for Cardiovascular Prevention (IPEK), Ludwig-Maximilians University Munich, Pettenkoferstr 9, 80336, Munich, Germany
[8] Mila - Quebec AI Institute, Montréal, Québec, Canada

Correspondence: daniel.taliun@mcgill.ca

Supplementary figures and tables

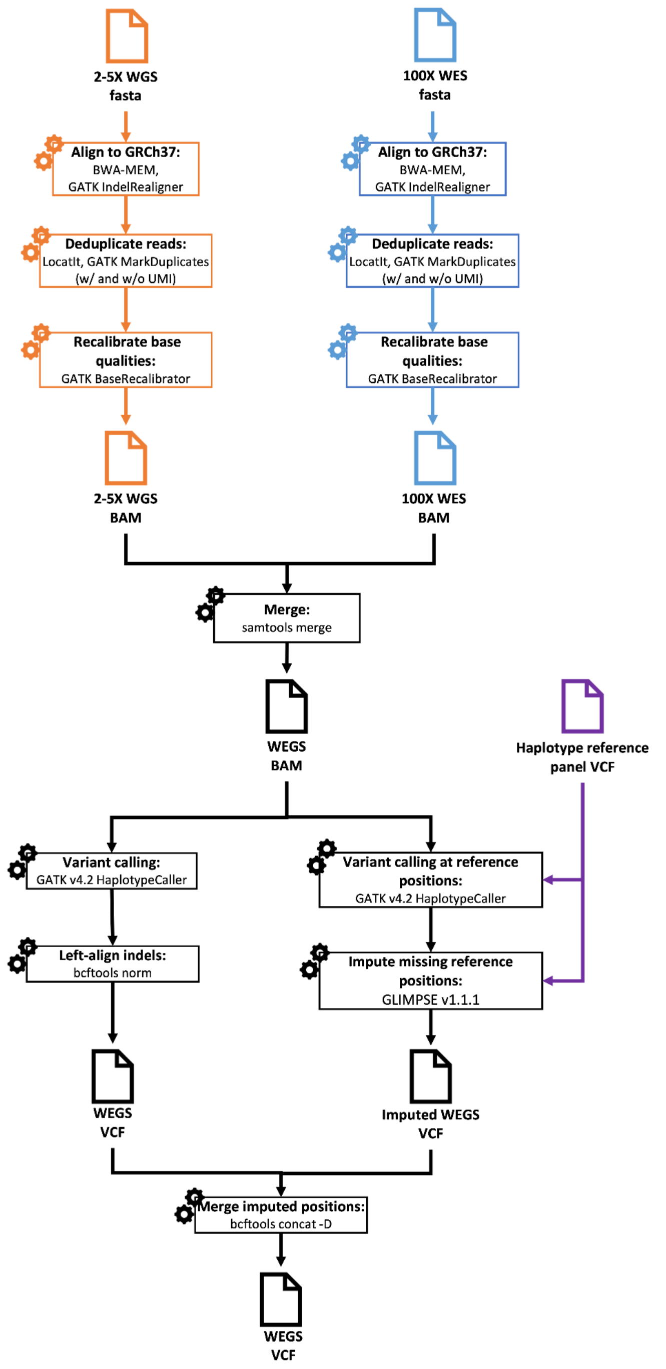

Supplementary Figure 1. Data processing diagram. WGS and WES alignment, deduplication and base recalibration steps were performed using GATK best practices. WGS and WES BAM files were then merged using samtools. Merged WEGS data was used for variant calling and imputation with GLIMPSE and local haplotype reference panel. The resulting imputed WEGS VCF was then used to merge imputed positions with WEGS data, hence obtaining the final WEGS VCF.

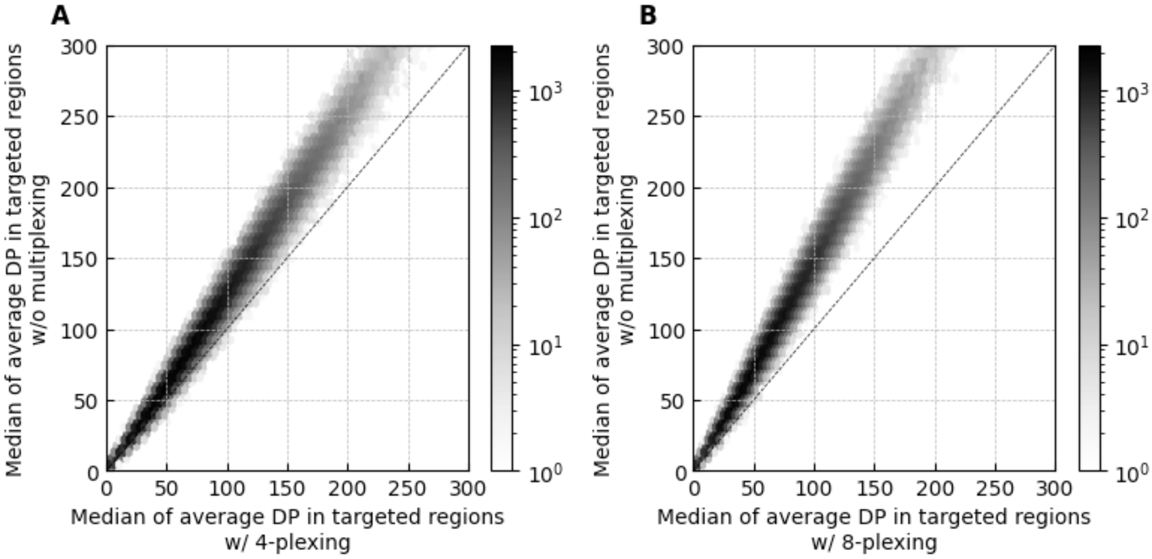

Supplementary Figure 2. Comparison of average depths of coverage (DP) in individual target regions in experiments without and with multiplexing. We computed the average depth of coverage (DP) for each target region in Agilent V7 capture in each sequenced individual. DP includes only paired mapped reads and base pairs with minimal Phred-scaled mapping and base qualities of 20. Each point corresponds to a single target region. In total, there were 208,817 non-overlapping autosomal regions. The X-axis shows the median of average DPs in the target region across all individuals sequenced in 4-plex (panel A) and 8-plex (panel B) experiments. The Y-axis shows the median of average DPs in the target region across all individuals sequenced without multiplexing. The darker color represents the higher density of the points. The vertical bar on the right of each panel shows the number of points corresponding to each color. The dotted black line corresponds to the 1:1 ratio between DP in the experiments.

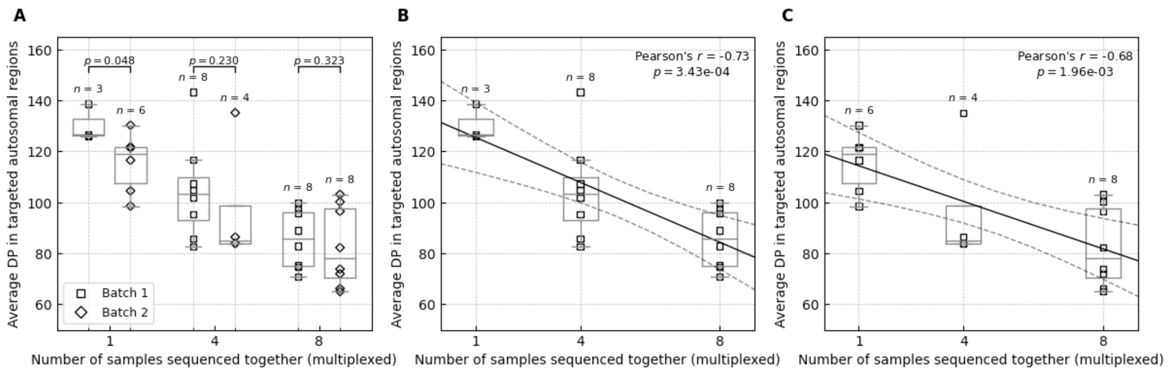

Supplementary Figure 3. Average depths of coverage across all targeted regions in autosomal chromosomes stratified by library preparation batch. The average depth of coverage (DP) was computed across target regions in Agilent V7 capture using paired mapped reads and counting only base pairs with minimal Phred-scaled mapping and base qualities of 20. The solid black line corresponds to the linear regression line, and the dashed black lines correspond to the 95% confidence interval. The box bounds the IQR, and Tukey-style whiskers extend to 1.5 × IQR beyond the box. The horizontal line within the box indicates the median value. Open rectangles and diamonds are data points corresponding to the average DP across individual exome in batches 1 and 2, respectively. A) The DP is stratified by the library preparation batch in experiments without multiplexing, with 4-plexing and 8-plexing experiments. The p-values above each experiment pair correspond to the one-tailed Wilcoxon rank-sum test. B) The DP in the first library preparation batch. C) The DP in the second library preparation batch.

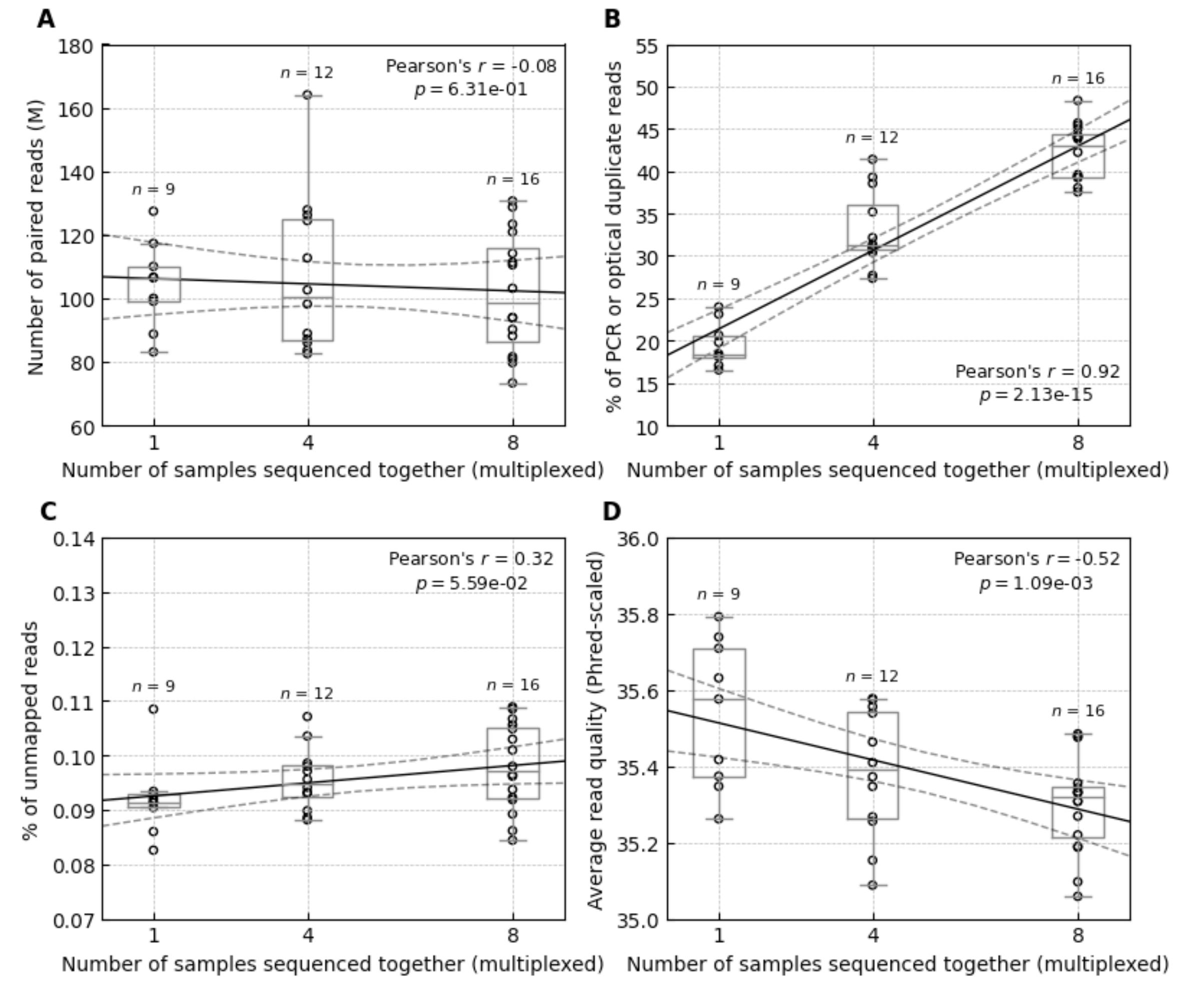

Supplementary Figure 4. Number of reads in autosomal chromosomes in sequencing experiments with and without multiplexing. The figure shows only paired reads in autosomal chromosomes, excluding reads that are non-primary or supplementary alignments or failed platform/vendor quality checks. The solid black line corresponds to the linear regression line, and the dashed black lines correspond to the 95% confidence interval. The box bounds the IQR, and Tukey-style whiskers extend to 1.5 × IQR beyond the box. The horizontal line within the box indicates the median value. Open circles are data points corresponding to the sequenced individual exomes. A) Number of paired reads in millions in sequencing experiments without sample multiplexing and when simultaneously sequencing four (4-plex) and eight (8-plex) samples. B) Percent of reads flagged as PCR or optical duplicates. C) Percent of unmapped reads. D) Average Phred-scaled base quality score across all reads in a sequenced sample.

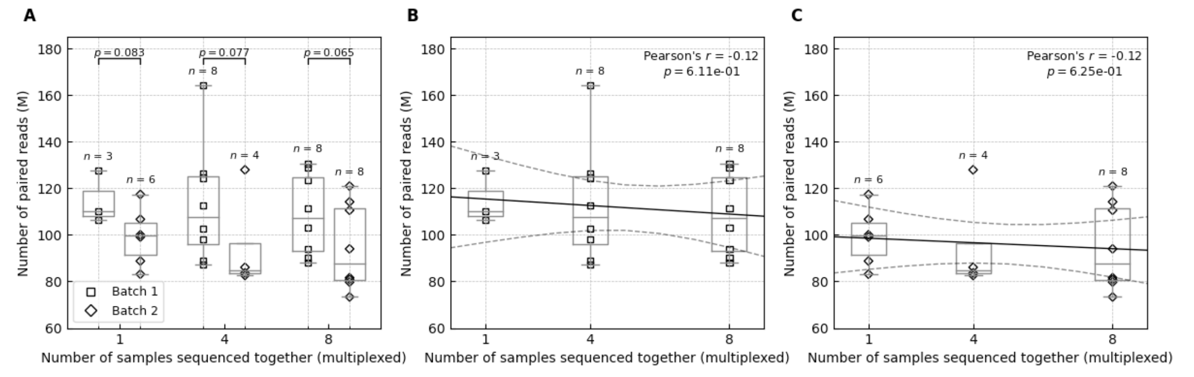

Supplementary Figure 5. The number of paired reads in autosomal chromosomes stratified by library preparation batch. The figure shows only paired reads in autosomal chromosomes, excluding reads that are non-primary or supplementary alignments or failed platform/vendor quality checks. The solid black line corresponds to the linear regression line, and the dashed black lines correspond to the 95% confidence interval. The box bounds the IQR, and Tukey-style whiskers extend to 1.5 × IQR beyond the box. The horizontal line within the box indicates the median value. Open rectangles and diamonds are data points corresponding to the number of paired reads across individual exome in batches 1 and 2, respectively. A) The number of paired reads is stratified by the library preparation batch in experiments without multiplexing, with 4-plexing and 8-plexing experiments. The p-values above each experiment pair correspond to the one-tailed Wilcoxon rank-sum test. B) The number of paired reads in the first library preparation batch. C) The number of paired reads in the second library preparation batch.

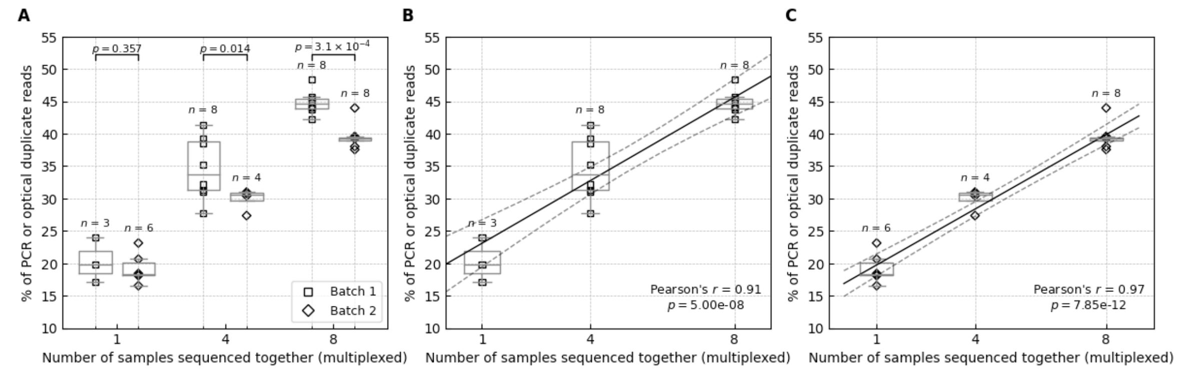

Supplementary Figure 6. Percent of reads flagged as PCR or optical duplicates in autosomal chromosomes stratified by library preparation batch. The figure shows only paired reads in autosomal chromosomes, excluding reads that are non-primary or supplementary alignments or failed platform/vendor quality checks. The solid black line corresponds to the linear regression line, and the dashed black lines correspond to the 95% confidence interval. The box bounds the IQR, and Tukey-style whiskers extend to 1.5 × IQR beyond the box. The horizontal line within the box indicates the median value. Open rectangles and diamonds are data points corresponding to the percent of duplicated reads across individual exomes in batches 1 and 2, respectively. A) The percent of duplicated reads is stratified by the library preparation batch in experiments without multiplexing, with 4-plexing and 8-plexing experiments. The p-values above each experiment pair correspond to the one-tailed Wilcoxon rank-sum test. B) The percent of duplicate reads in the first library preparation batch. C) The percent of duplicate reads in the second library preparation batch.

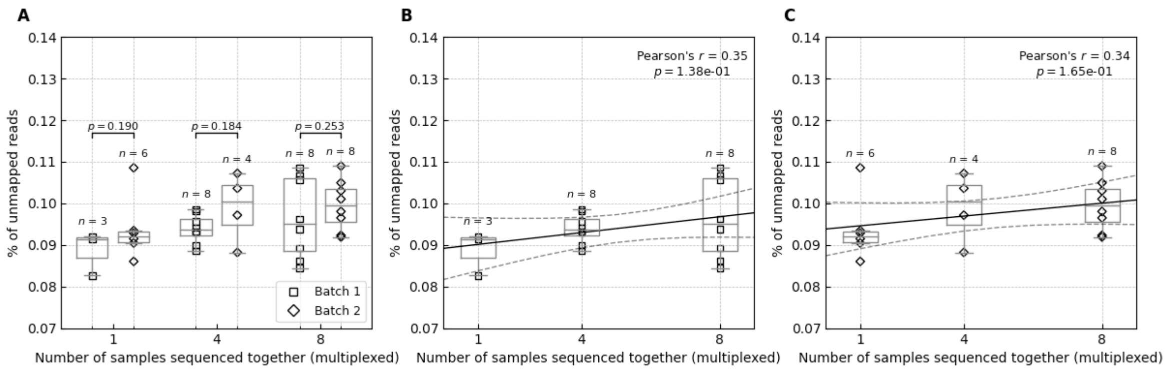

Supplementary Figure 7. Percent of unmapped reads in autosomal chromosomes stratified by library preparation batch. The figure shows only paired reads in autosomal chromosomes, excluding reads that are non-primary or supplementary alignments or failed platform/vendor quality checks. The solid black line corresponds to the linear regression line, and the dashed black lines correspond to the 95% confidence interval. The box bounds the IQR, and Tukey-style whiskers extend to 1.5 × IQR beyond the box. The horizontal line within the box indicates the median value. Open rectangles and diamonds are data points corresponding to the percent of unmapped reads across individual exome in batches 1 and 2, respectively. A) The percent of unmapped reads is stratified by the library preparation batch in experiments without multiplexing, with 4-plexing and 8-plexing experiments. The p-values above each experiment pair correspond to the one-tailed Wilcoxon rank-sum test. B) The percent of unmapped reads in the first library preparation batch. C) The percent of unmapped reads in the second library preparation batch.

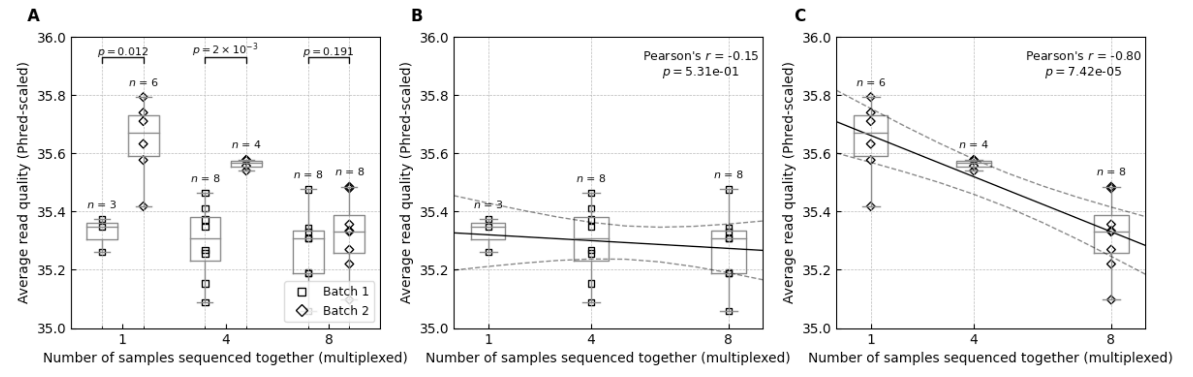

Supplementary Figure 8. The average quality of reads in autosomal chromosomes stratified by library preparation batch. The figure shows only paired reads in autosomal chromosomes, excluding reads that are non-primary or supplementary alignments or failed platform/vendor quality checks. The average read quality was computed as the average of Phred-scaled base qualities. The solid black line corresponds to the linear regression line, and the dashed black lines correspond to the 95% confidence interval. The box bounds the IQR, and Tukey-style whiskers extend to 1.5 × IQR beyond the box. The horizontal line within the box indicates the median value. Open rectangles and diamonds are data points corresponding to the average read quality across individual exome in batches 1 and 2, respectively. A) The average read quality is stratified by the library preparation batch in experiments without multiplexing, with 4-plexing and 8-plexing experiments. The p-values above each experiment pair correspond to the one-tailed Wilcoxon rank-sum test. B) The average read quality in the first library preparation batch. C) The average read quality in the second library preparation batch.

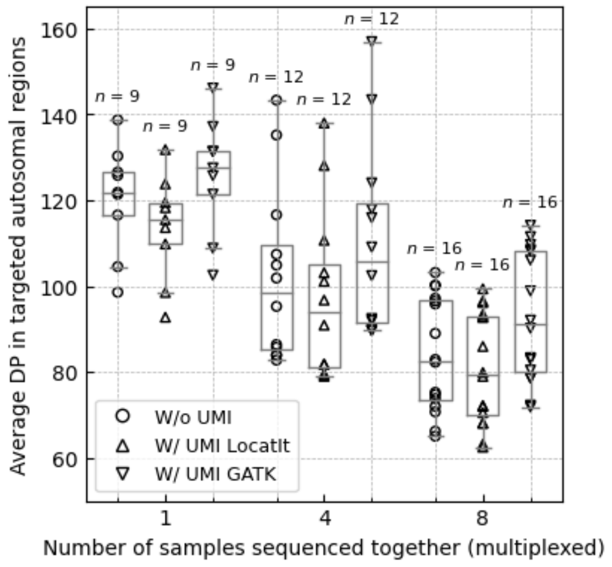

Supplementary Figure 9. Average depths of coverage across all targeted regions in autosomal chromosomes processed with and without UMI-aware deduplication. The average depth of coverage (DP) was computed across target regions in Agilent V7 capture using paired mapped reads and counting only base-pairs with minimal Phred-scaled mapping and base qualities of 20. The box bounds the IQR and Tukey-style whiskers extend to a maximum of 1.5 × IQR beyond the box. The horizontal line within the box indicates median value. Open circles, up-pointing and down-pointing triangles are data points corresponding to the average DP across individual exome processed without, with LocatIt and GATK's UmiAwareMarkDuplicatesWithMateCigar UMI-aware deduplication, respectively.

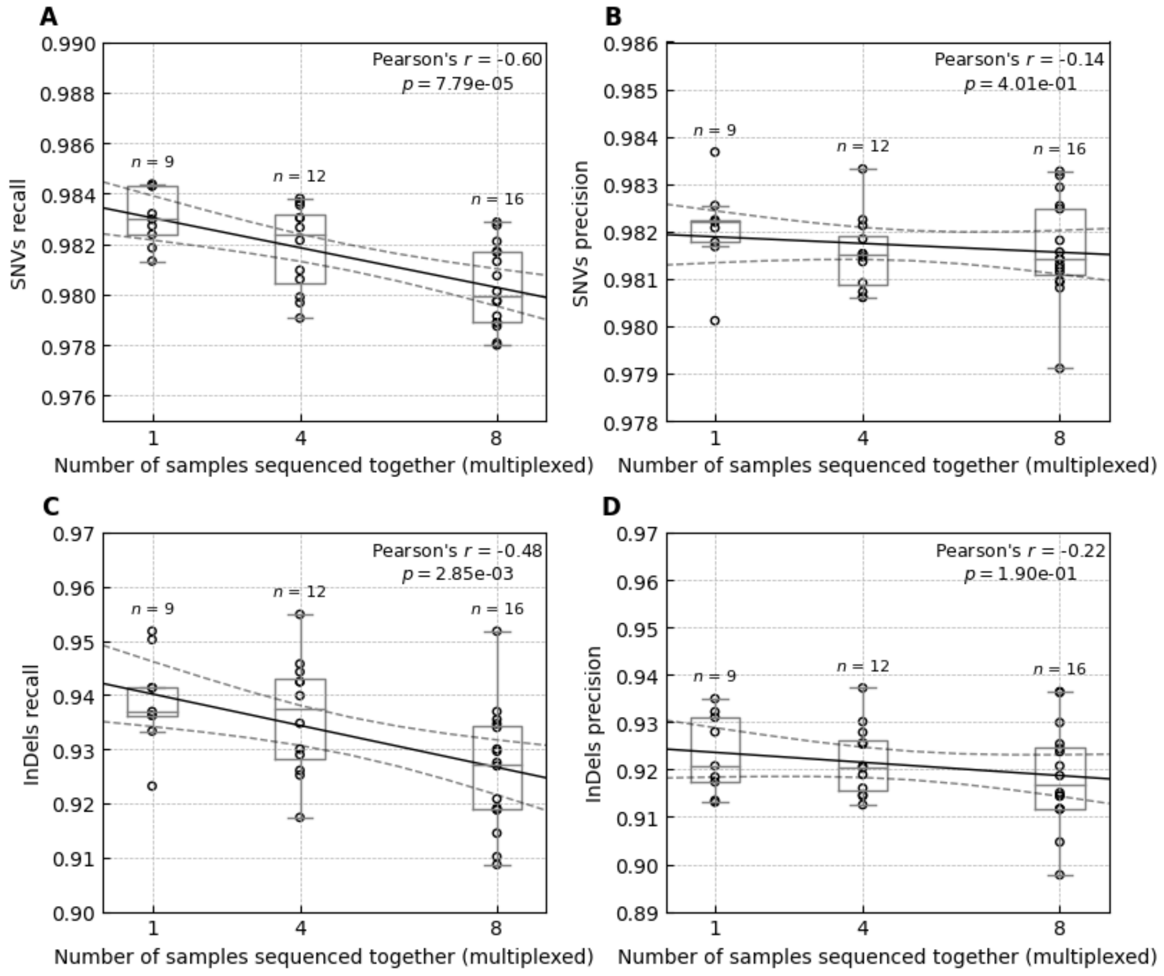

Supplementary Figure 10. Recall and precision of the SNVs and InDels called in sequencing experiments without and with multiplexing. The figure represents variant calls inside the target regions in Agilent V7 capture and the GIAB high-confidence regions. The solid black line corresponds to the linear regression line, and the dashed black lines correspond to the 95% confidence interval. The box bounds the IQR, and Tukey-style whiskers extend to 1.5 × IQR beyond the box. The horizontal line within the box indicates the median value. Open circles are data points corresponding to the sequenced individual exomes. A) Recall rates of the called SNVs. B) Precision of the called SNVs. C) Recall rates of the called InDels. D) Precision of the called InDels.

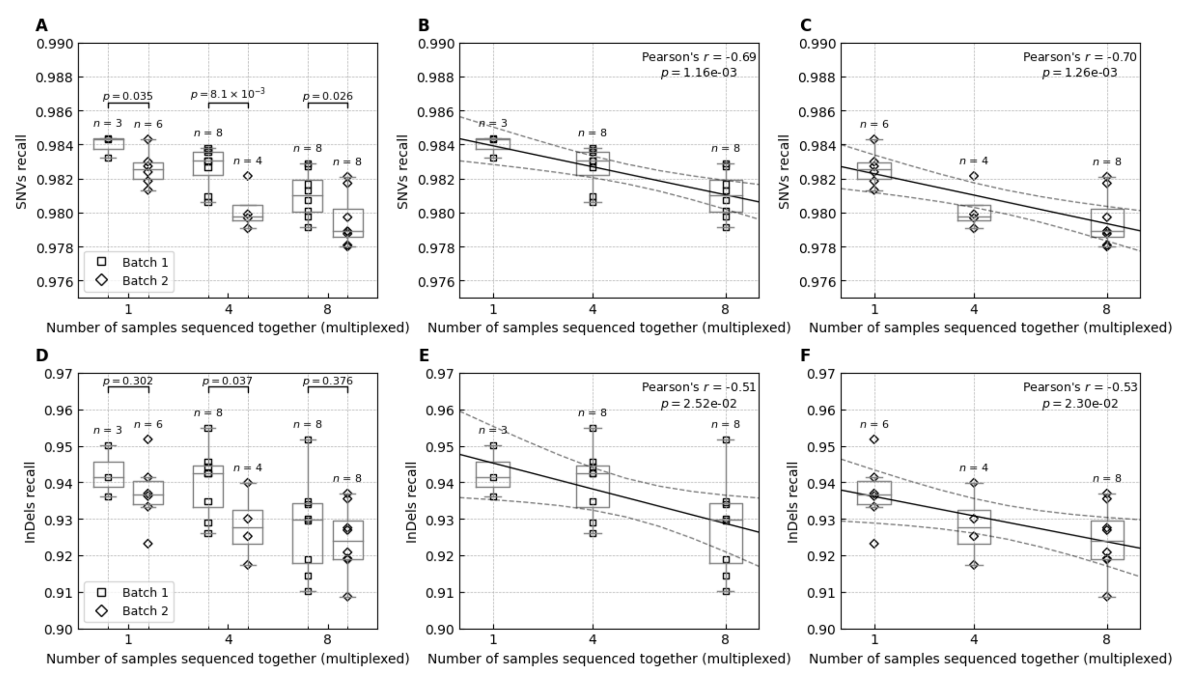

Supplementary Figure 11. The recall of the SNVs and InDels called in sequencing experiments without and with multiplexing stratified by library preparation batch. The figure represents variant calls inside the target regions in Agilent V7 capture and the GIAB high-confidence regions. The solid black line corresponds to the linear regression line, and the dashed black lines correspond to the 95% confidence interval. The box bounds the IQR, and Tukey-style whiskers extend to 1.5 × IQR beyond the box. The horizontal line within the box indicates the median value. Open rectangles and diamonds are data points corresponding to the recall across individual exome in batches 1 and 2, respectively. A) The recall of SNVs is stratified by the library preparation batch in experiments without multiplexing, with 4-plexing and 8-plexing experiments. The p-values above each experiment pair correspond to the one-tailed Wilcoxon rank-sum test. B) The recall of SNVs in the first library preparation batch. C) The recall of SNVs in the second library preparation batch. D) The recall of InDels is stratified by the library preparation batch in experiments without multiplexing, with 4-plexing and 8-plexing experiments. The p-values above each experiment pair correspond to the one-tailed Wilcoxon rank-sum test. E) The recall of InDels in the first library preparation batch. F) The recall of InDels in the second library preparation batch.

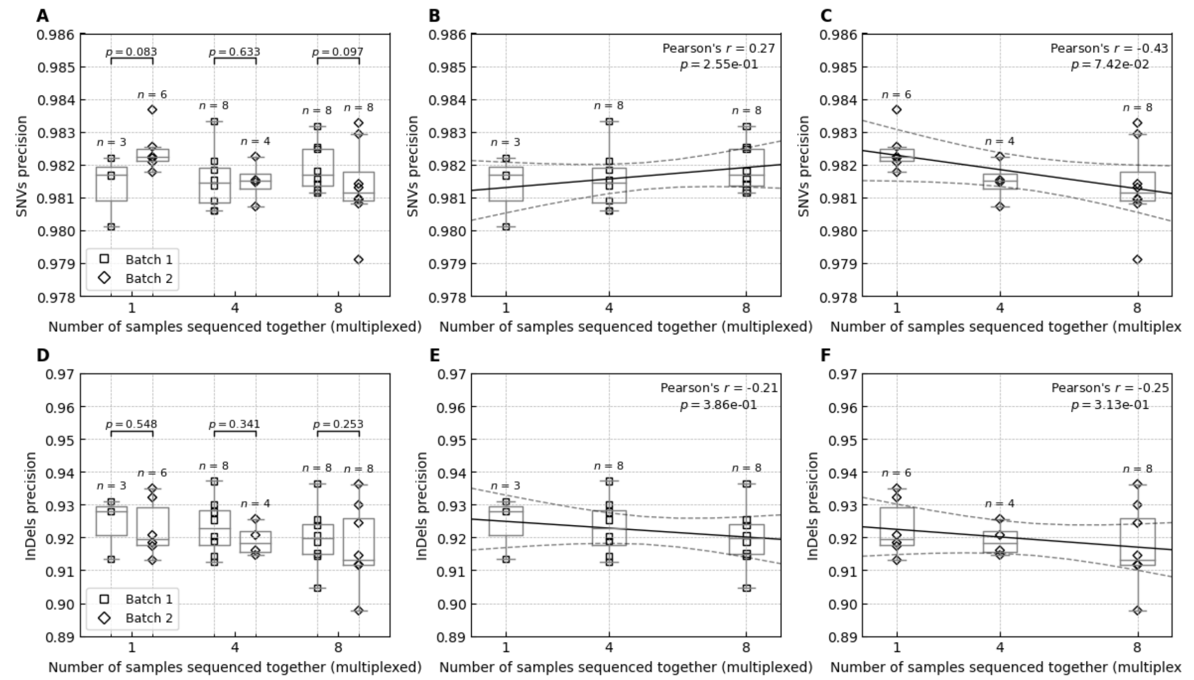

Supplementary Figure 12. The precision of the SNVs and InDels called in sequencing experiments without and with multiplexing stratified by library preparation batch. The figure represents variant calls inside the target regions in Agilent V7 capture and the GIAB high-confidence regions. The solid black line corresponds to the linear regression line, and the dashed black lines correspond to the 95% confidence interval. The box bounds the IQR, and Tukey-style whiskers extend to 1.5 × IQR beyond the box. The horizontal line within the box indicates the median value. Open rectangles and diamonds are data points corresponding to the precision across individual exome in batches 1 and 2, respectively. A) The precision of SNVs is stratified by the library preparation batch in experiments without multiplexing, with 4-plexing and 8-plexing experiments. The p-values above each experiment pair correspond to the one-tailed Wilcoxon rank-sum test. B) The precision of SNVs in the first library preparation batch. C) The precision of SNVs in the second library preparation batch. D) The precision of InDels is stratified by the library preparation batch in experiments without multiplexing, with 4-plexing and 8-plexing experiments. The p-values above each experiment pair correspond to the one-tailed Wilcoxon rank-sum test. E) The precision of InDels in the first library preparation batch. F) The precision of InDels in the second library preparation batch.

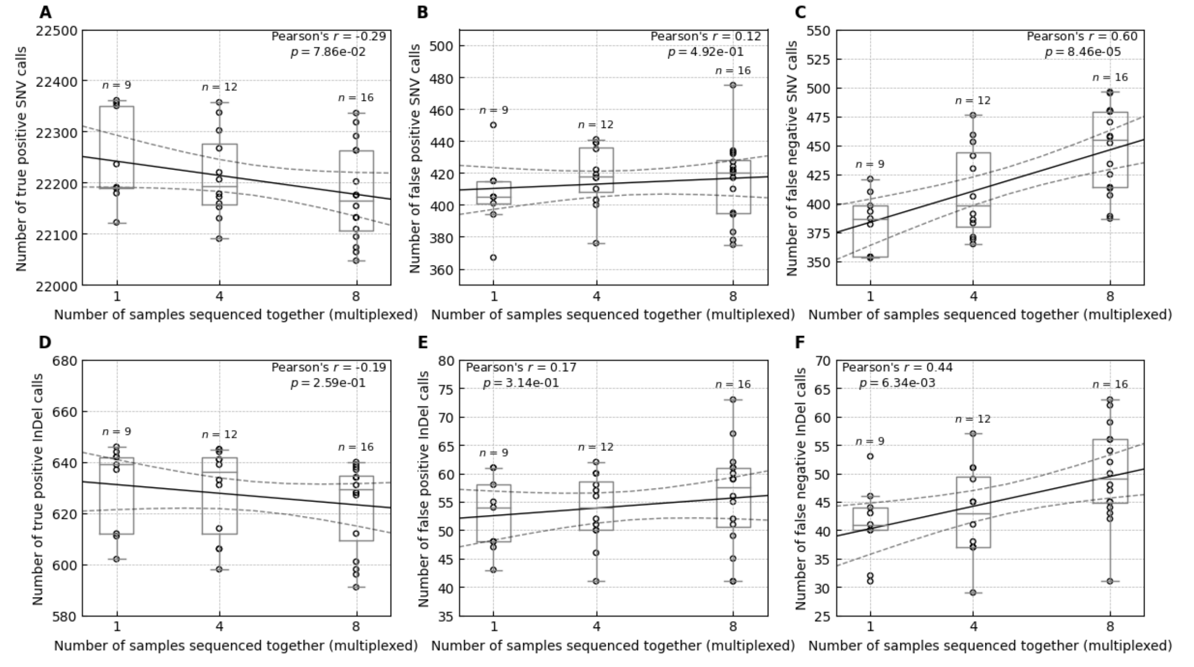

Supplementary Figure 13. The number of SNV and InDel calls in sequencing experiments without and with multiplexing. The figure represents variant calls inside the target regions in Agilent V7 capture and the GIAB high-confidence regions. The solid black line corresponds to the linear regression line, and the dashed black lines correspond to 95% confidence interval. The box bounds the IQR, and Tukey-style whiskers extend to 1.5 × IQR beyond the box. The horizontal line within the box indicates the median value. A) Number of true positive (TP) SNV calls in sequencing experiments without sample multiplexing and when simultaneously sequencing four (4-plex) and eight (8-plex) samples. B) Number of false positive (FP) SNV calls. C) Number of false negative (FN) SNV calls. D) Number of TP InDel calls. E) Number FP InDel calls. F) Number of FN InDel calls.

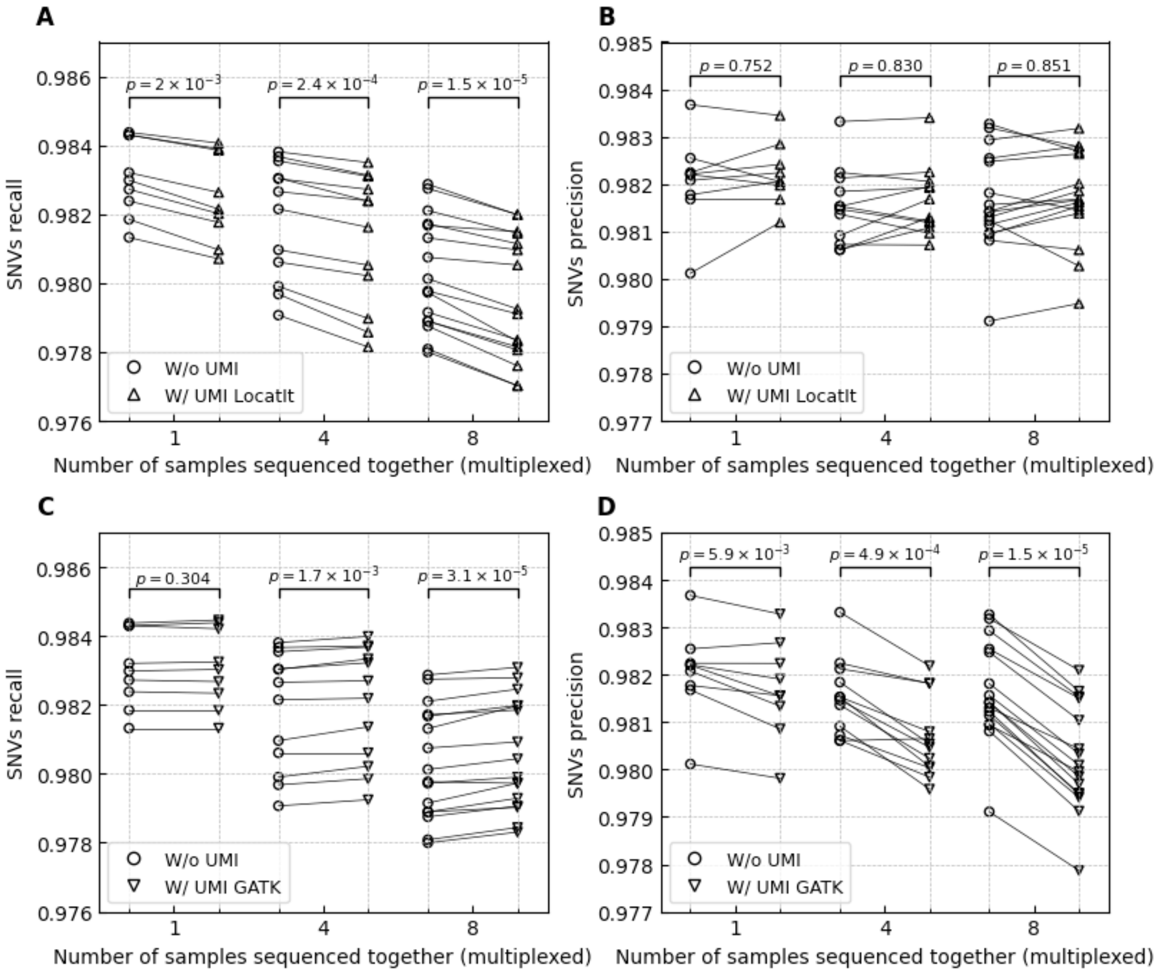

Supplementary Figure 14. Recall and precision of the single nucleotide variations (SNVs) in sequencing experiments without and with UMI-aware read deduplication. The figure represents SNV calls inside the target regions in Agilent V7 capture and the GIAB high-confidence regions. Open circles, up-pointing and down-pointing triangles are data points corresponding to the recall and precision in individual exomes processed without, with LocatIt and GATK's UmiAwareMarkDuplicatesWithMateCigar UMI-aware deduplication, respectively. The solid black lines connect pairs of individual exomes with the same underlying sequencing data (i.e. same sequenced sample) but different deduplication approaches. The p-values above experiments with varying levels of multiplexing correspond to the one-tailed Wilcoxon signed-rank test between UMI agnostic and UMI-aware deduplication. A) Recall rates of the called SNVs without UMI-aware compared to UMI-aware deduplication using LocatIt. B) Precision of the called SNVs without UMI-aware compared to UMI-aware deduplication using LocatIt. C) Recall rates of the called SNVs without UMI-aware compared to UMI-aware deduplication using GATK's UmiAwareMarkDuplicatesWithMateCigar. D) Precision of the called SNVs without UMI-aware compared to UMI-aware deduplication using GATK's UmiAwareMarkDuplicatesWithMateCigar.

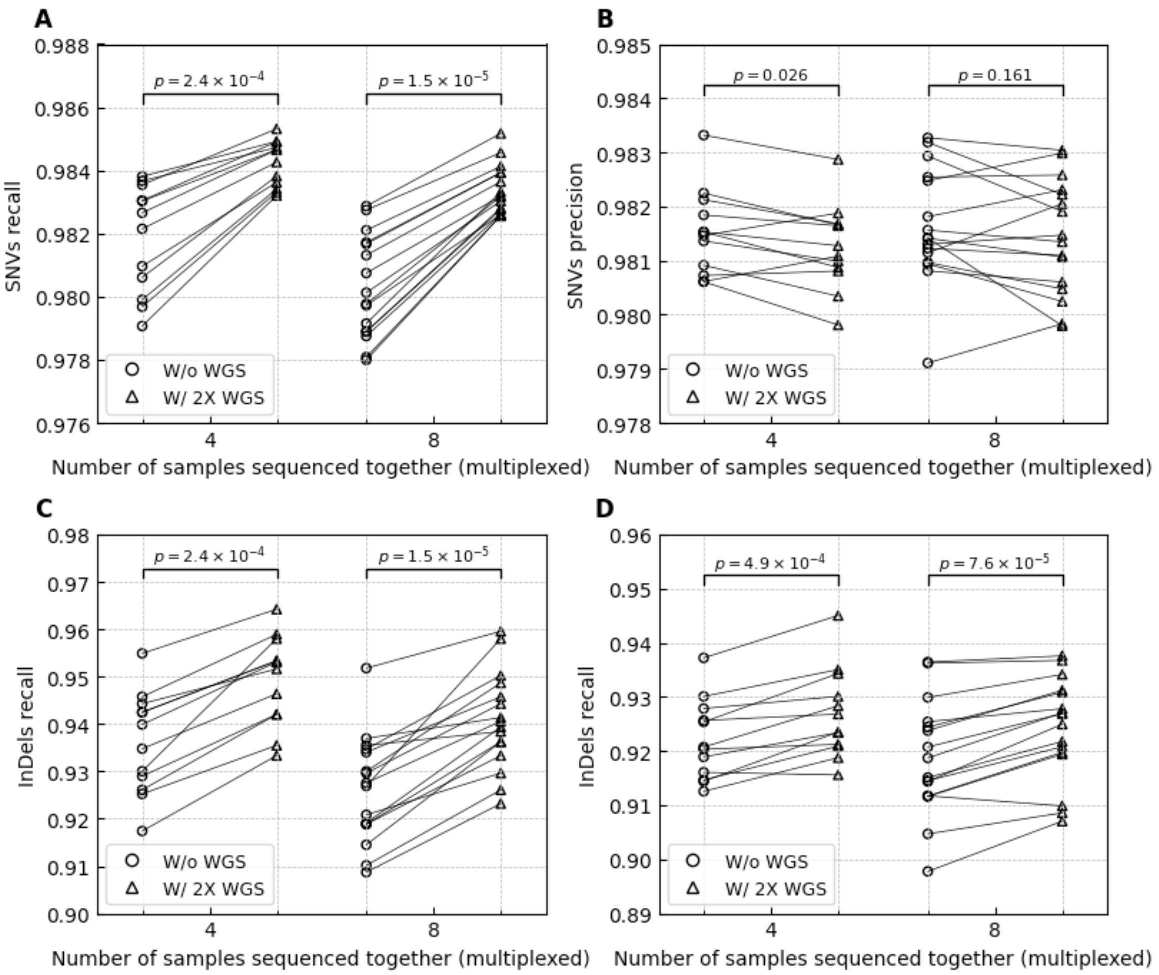

Supplementary Figure 15. Variant recall and precision rates in WES experiments with multiplexing before and after adding 2X WGS data. The figure represents variant calls inside the target regions in Agilent V7 capture and the GIAB high-confidence regions. Open circles and up-pointing triangles are data points corresponding to the recall and precision in individual multiplexed WES before and after adding 2X WGS data, respectively. The solid black lines connect pairs of individual exomes with the same underlying WES data (i.e. same sequenced sample). The p-values above experiments with varying levels of multiplexing correspond to the one-tailed Wilcoxon signed-rank test between UMI agnostic and UMI-aware deduplication. A) Recall rates of the called SNVs with and without 2X WGS. B) Precision rates of the called SNVs with and without 2X WGS. C) Recall rates of the called InDels with and without 2X WGS. D) Precision rates of the called InDels with and without 2X WGS.

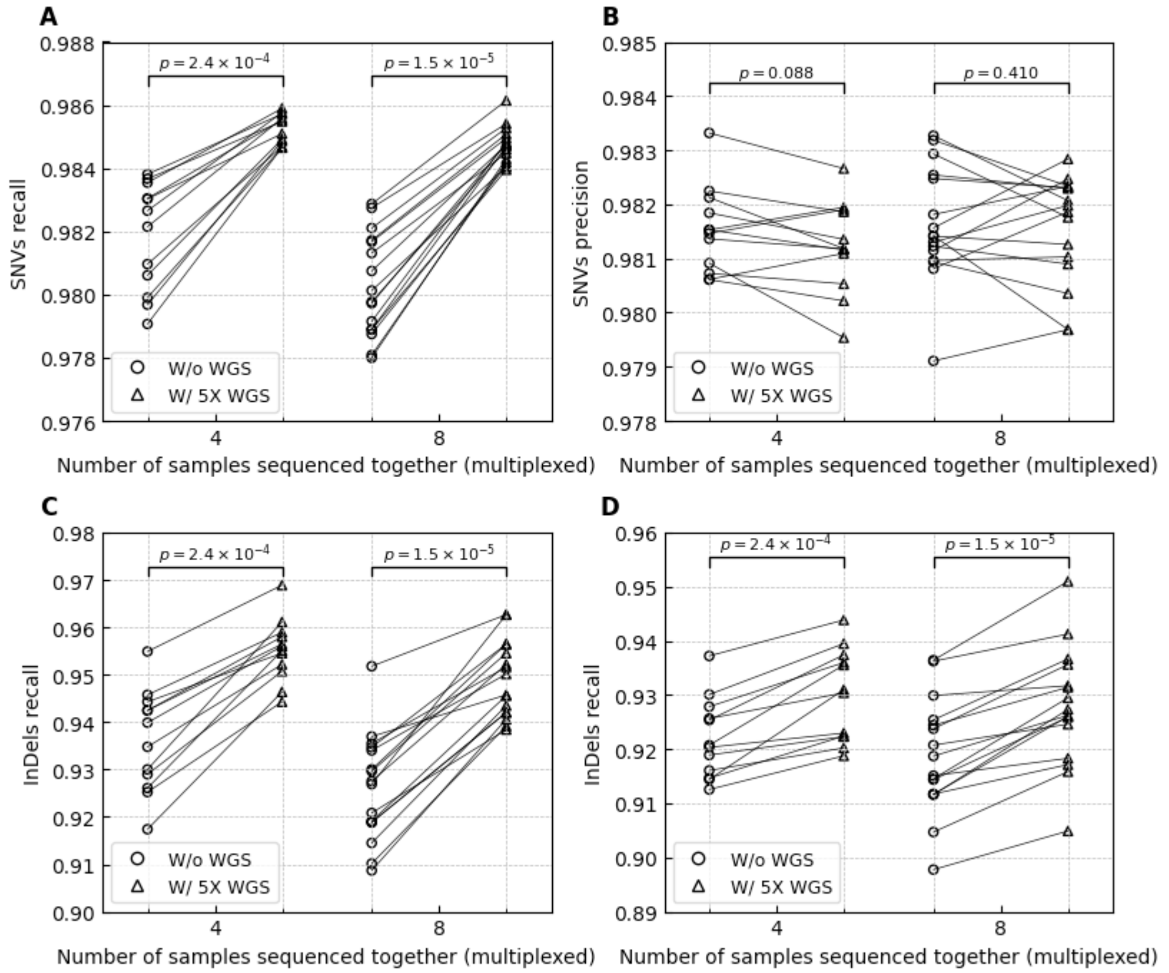

Supplementary Figure 16. Variant recall and precision rates in WES experiments with multiplexing before and after adding 5X WGS data. The figure represents variant calls inside the target regions in Agilent V7 capture and the GIAB high-confidence regions. Open circles and up-pointing triangles are data points corresponding to the recall and precision in individual multiplexed WES before and after adding 5X WGS data, respectively. The solid black lines connect pairs of individual exomes with the same underlying WES data (i.e. same sequenced sample). The p-values above experiments with varying levels of multiplexing correspond to the one-tailed Wilcoxon signed-rank test between UMI agnostic and UMI-aware deduplication. A) Recall rates of the called SNVs with and without 5X WGS. B) Precision rates of the called SNVs with and without 5X WGS. C) Recall rates of the called InDels with and without 5X WGS. D) Precision rates of the called InDels with and without 5X WGS.

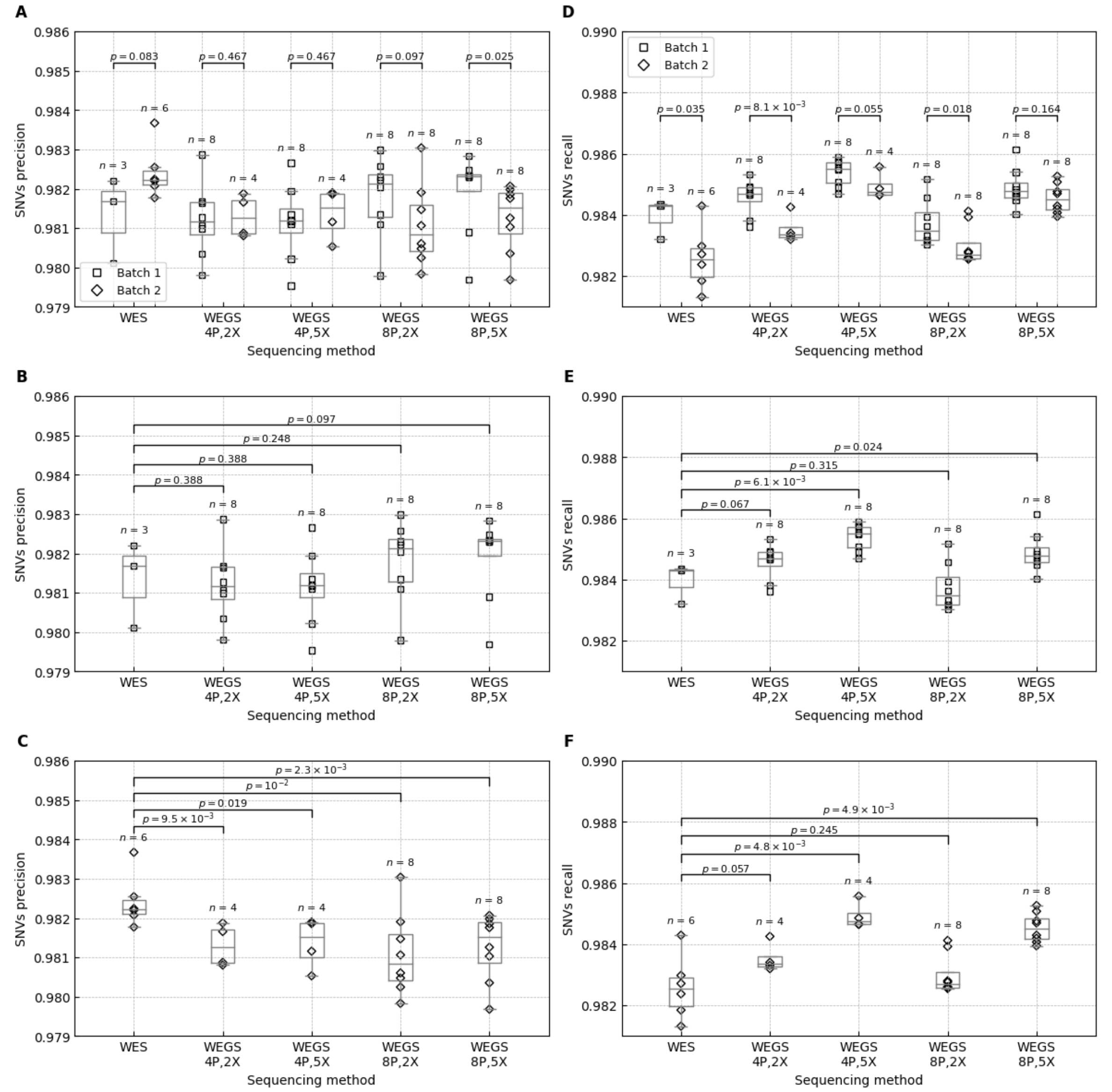

Supplementary Figure 17. SNVs calling precision and recall rates in no-plexing WES compared to WEGS stratified by library preparation batch. The figure represents SNV calls inside the target regions in Agilent V7 capture and the GIAB high-confidence regions. The box bounds the IQR, and Tukey-style whiskers extend to 1.5 × IQR beyond the box. The horizontal line within the box indicates the median value. Open rectangles and diamonds are data points corresponding to the individual WES and WEGS in batches 1 and 2, respectively. The p-values above each pair of batches or sequencing methods correspond to the one-tailed Wilcoxon rank-sum test. A) Precision rates of the called SNVs in batches 1 and 2. B) Precision rates of the called SNVs in batch 1. C) Precision rates of the called SNVs in batch 2. D) Recall rates of the called SNVs in batches 1 and 2. E) Recall rates of the called SNVs in batch 1. F) Recall rates of the called SNVs in batch 2. Supplementary Table 7 shows average values and standard errors.

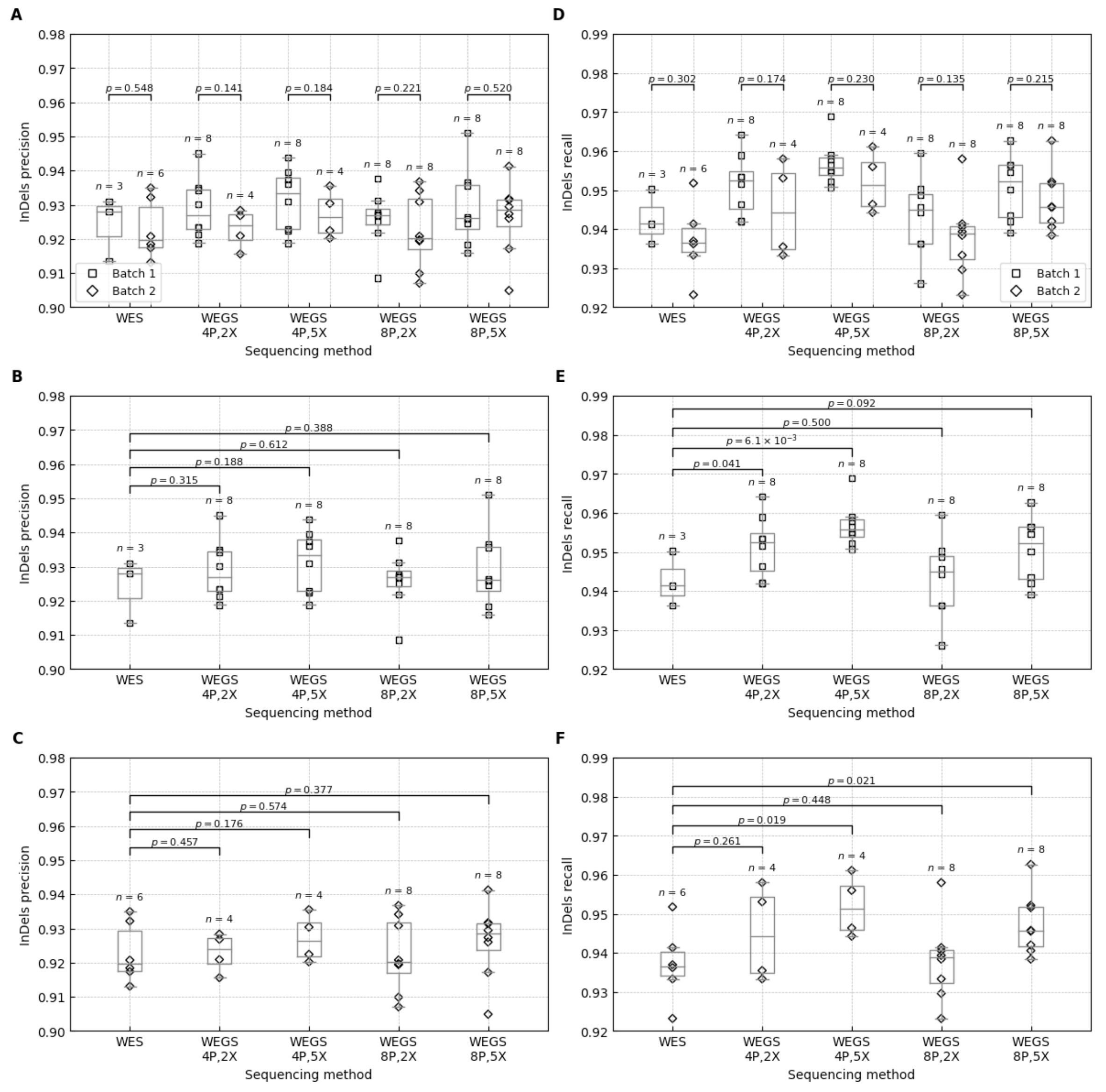

Supplementary Figure 18. InDel calling precision and recall rates in no-plexing WES compared to WEGS stratified by library preparation batch. The figure represents InDel calls inside the target regions in Agilent V7 capture and the GIAB high-confidence regions. The box bounds the IQR, and Tukey-style whiskers extend to 1.5 × IQR beyond the box. The horizontal line within the box indicates the median value. Open rectangles and diamonds are data points corresponding to the individual WES and WEGS in batches 1 and 2, respectively. The p-values above each pair of batches or sequencing methods correspond to the one-tailed Wilcoxon rank-sum test. A) Precision rates of the called InDels in batches 1 and 2. B) Precision rates of the called InDels in batch 1. C) Precision rates of the called InDels in batch 2. D) Recall rates of the called InDels in batches 1 and 2. E) Recall rates of the called InDels in batch 1. F) Recall rates of the called InDels in batch 2. Supplementary Table 7 shows average values and standard errors.

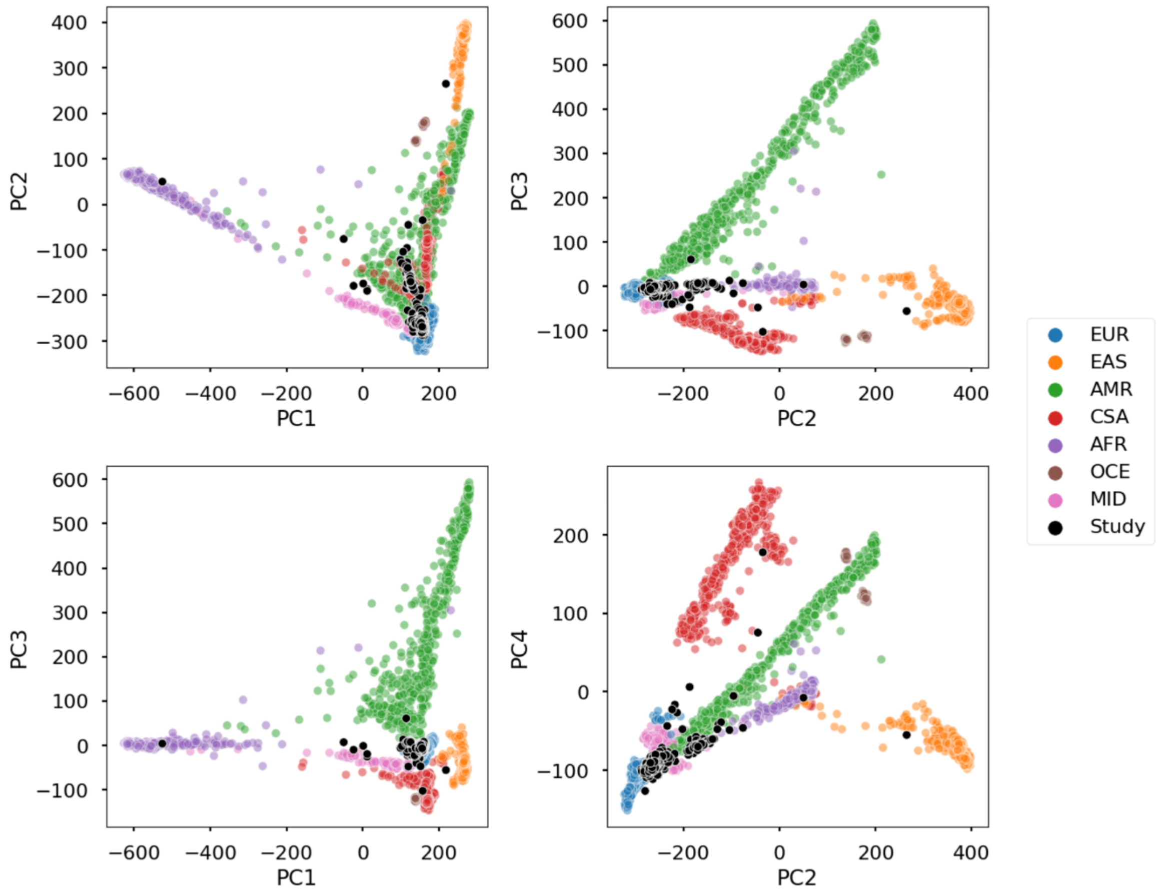

Supplementary Figure 19. PCA projection of the sequenced PAD patients into the 1000 Genomes Project (1kGP) and Human Genome Diversity Project (HGDP) combined reference panel. Open black circles represent sequenced individuals. Colored circles represent reference individuals from the 1kGP/HGDP dataset.

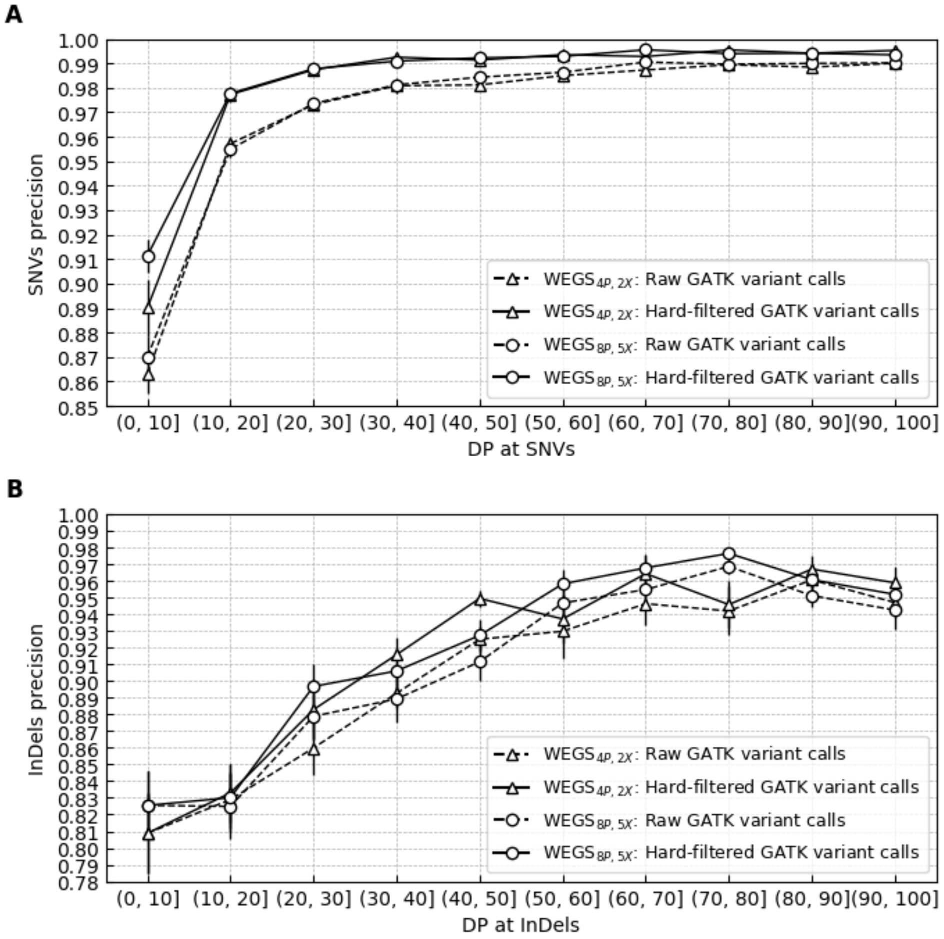

Supplementary Figure 20. Precision of variant calls in WEGS targeted regions in GIAB samples stratified by depth of coverage (DP) and variant filtering. The figure represents SNV and InDel calls inside the target regions in Agilent V7 capture and the GIAB high-confidence regions. Shapes represent the average values, while vertical lines represent standard errors. For InDels, DP at the start position of InDel variant was used.

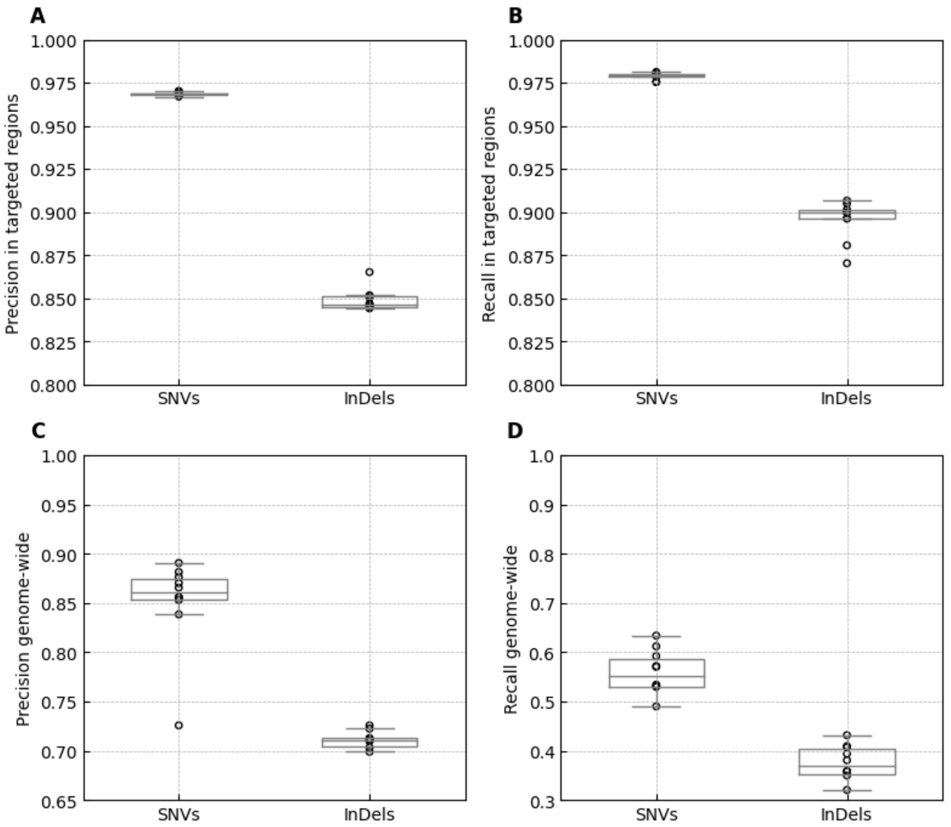

Supplementary Figure 21. Precision and recall of variant calls in ten replicates of HG002 control sample in PAD study when using WEGS$_{8P,4X}$. The HG002 sample was used as a control during the sequencing of PAD samples, which resulted in 10 replicates. We calculated the variant recall and precision rates for each HG002 replicate against the GIAB reference data. The box bounds the IQR, and Tukey-style whiskers extend to 1.5 × IQR beyond the box. The horizontal line within the box indicates the median value. An open circle represents the precision or recall values for an individual HG002 replicate. Supplementary Table 1 lists the mean values and standard errors.

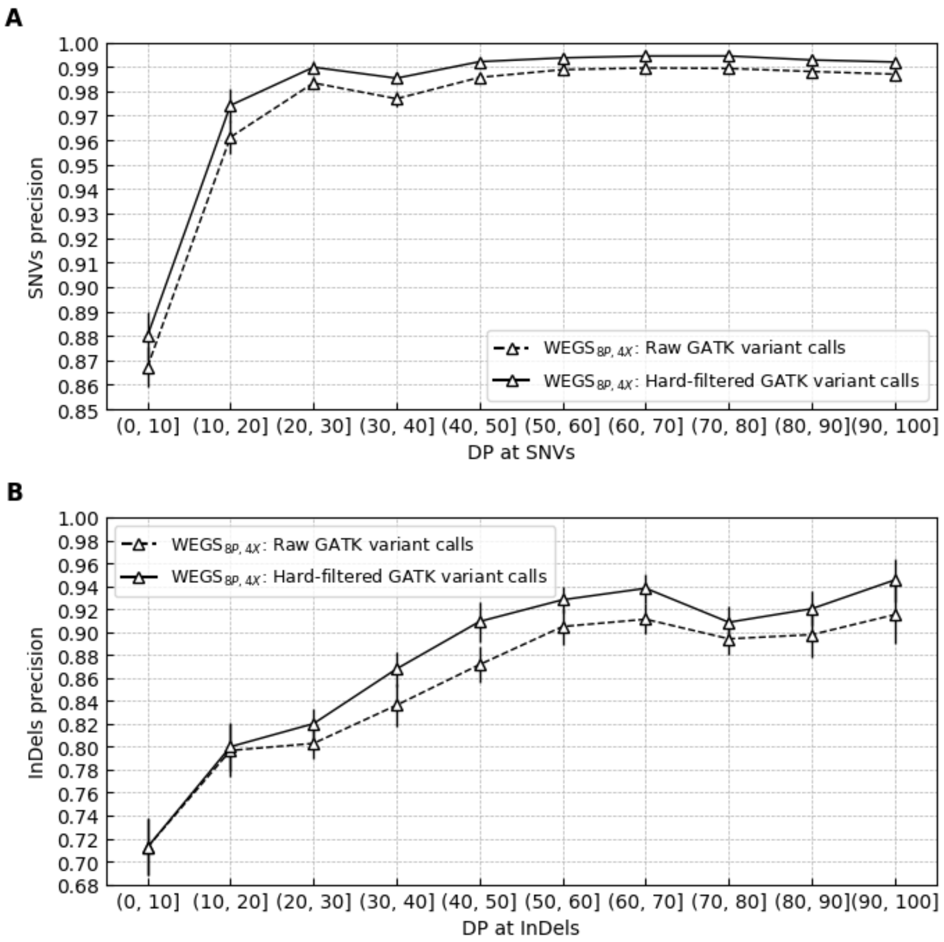

Supplementary Figure 22. Precision of variant calls in targeted regions in ten replicates of HG002 control sample in PAD study when using WEGS8P,4X stratified by depth of coverage (DP). The figure represents SNV and InDel calls inside the target regions in Agilent V7 capture and the GIAB high-confidence regions. The HG002 sample was used as a control during the sequencing of PAD samples, which resulted in 10 replicates. We calculated the variant precision rates for each HG002 replicate against the GIAB reference data. Shapes represent the average values, while vertical lines represent standard errors. For InDels, DP at the start position of InDel was used.

Supplementary Table 1. Precision and recall of variant calls in ten replicates of HG002 control sample in PAD study when using WEGS$_{8P,4X}$. The HG002 sample was used as a control during the sequencing of PAD samples, which resulted in 10 replicates. We calculated the variant recall and precision rates for each HG002 replicate against the GIAB reference data.

Category	SNVs Recall (SE)	SNVs Precision (SE)	InDel Recall (SE)	InDel Precision (SE)
Target regions	0.9788 (0.0006)	0.9685 (0.0003)	0.8955 (0.0036)	0.8490 (0.0020)
Genome-wide	0.5274 (0.0379)	0.8513 (0.0148)	0.3515 (0.0293)	0.7028 (0.0094)

Supplementary Table 2. The average changes in read properties after UMI-aware read deduplication steps relative to the UMI agnostic approach.

Number of samples sequenced together (multiplexed)	UMI-aware deduplication tool	Average difference compared to UMI agnostic approach			
		% of QC fail reads (SE)	% of PCR/optical duplicates (SE)	% of unmapped reads (SE)	Avg. Phred-scaled read quality (SE)
1	LocatIt	6.45 (0.16)	-1.20 (0.04)	-0.068 (0.00223)	2.60 (0.12)
1	GATK	0.00 (0.00)	-0.36 (0.01)	0.003 (0.00003)	0.35 (0.06)
4	LocatIt	5.19 (0.16)	-1.42 (0.04)	-0.069 (0.00159)	2.87 (0.05)
4	GATK	0.00 (0.00)	-0.39 (0.01)	0.003 (0.00005)	0.49 (0.05)
8	LocatIt	4.38 (0.10)	-1.56 (0.03)	-0.069 (0.00175)	2.70 (0.14)
8	GATK	0.00 (0.00)	-0.40 (0.01)	0.003 (0.00004)	0.57 (0.03)

Supplementary Table 3. Variant calling in whole exome sequencing experiments with and without multiplexing. The table represents variant calls inside the target regions in Agilent V7 capture and the GIAB high-confidence regions. TP - true positives, FP - false positives, FN - false negatives.

	SNVs						InDels					
Number of samples sequenced together (multiplexed)	N (SE)	TP (SE)	FP (SE)	FN (SE)	Recall (SE)	Precision (SE)	N (SE)	TP (SE)	FP (SE)	FN (SE)	Recall (SE)	Precision (SE)
1	22,648 (33)	22,241 (30)	406 (7)	384 (8)	0.9830 (0.0004)	0.9821 (0.0003)	683 (7)	631 (6)	53 (2)	41 (2)	0.9390 (0.0029)	0.9232 (0.0028)
4	22,632 (28)	22,214 (24)	418 (6)	411 (11)	0.9818 (0.0005)	0.9815 (0.0002)	682 (7)	629 (5)	54 (2)	43 (2)	0.9360 (0.0031)	0.9220 (0.0021)
8	22,592 (28)	22,177 (23)	415 (6)	446 (9)	0.9803 (0.0004)	0.9816 (0.0003)	679 (6)	623 (4)	56 (2)	50 (2)	0.9261 (0.0028)	0.9186 (0.0027)

Supplementary Table 4. The average number of SNVs missed in multiplexing experiments but correctly identified across all no-plexing experiments. For each multiplexing experiment, we computed the number of false negative (FN) SNV calls that were true positive (TP) in all three no-plexing experiments for the corresponding individual.

	TP across all no-plexing WES	
	All N (SE)	Higher DP N (SE)
**FN in 4-plexing WES**	45 (6)	40 (6)
**FN in 8-plexing WES**	65 (6)	61 (6)

Supplementary Table 5. The average changes in SNV calling in whole exome sequencing experiments with UMI-aware read deduplication relative to the UMI agnostic approach. The table represents SNV calls inside the target regions in Agilent V7 capture and the GIAB high-confidence regions. The star symbols represent statistically significant differences when using a one-tailed Wilcoxon signed-rank test: * - P-value < 0.05, ** - P-value < 0.01, *** - P-value < 0.001.

Number of samples sequenced together (multiplexed)	UMI-aware deduplication tool	N (SE)	TP (SE)	FP (SE)	FN (SE)	Recall (SE)	Precision (SE)
1	LocatIt	-17 (3) **	-14 (1) **	-4 (4)	14 (1) **	-0.0006 (0.0001) **	0.0002 (0.0002)
1	GATK	9 (3) **	0 (0)	8 (2) **	-0 (0)	0.0000 (0.0000)	-0.0004 (0.0001)**
4	LocatIt	-16 (3) ***	-13 (2) ***	-3 (3)	13 (2) ***	-0.0006 (0.0001)***	0.0001 (0.0001)
4	GATK	24 (3) ***	4 (1) **	20 (3) ***	-4 (1) **	0.0002 (<0.0001) **	-0.0009 (0.0001)***
8	LocatIt	-20 (4) ***	-17 (2) ***	-2 (3)	17 (2) ***	-0.0008 (0.0001)***	0.0001 (0.0001)
8	GATK	39 (2) ***	6 (1) ***	33 (2) ***	-6 (1) ***	0.0003 (<0.0001)***	-0.0014 (0.0001)***

Supplementary Table 6. The average changes in SNVs and InDels calling in whole exome sequencing experiments when adding additional whole genome sequencing reads relative to pure whole exome sequencing experiments. The table represents SNV and InDel calls inside the target regions in Agilent V7 capture and the GIAB high-confidence regions. The star symbols represent statistically significant differences when using a one-tailed Wilcoxon signed-rank test: * - P-value < 0.05, ** - P-value < 0.01, *** - P-value < 0.001.

Number of samples sequenced together (multiplexed)	WGS DP	SNVs						InDels					
		N (SE)	TP (SE)	FP (SE)	FN (SE)	Recall (SE)	Precision (SE)	N (SE)	TP (SE)	FP (SE)	FN (SE)	Recall (SE)	Precision (SE)
4	2X	62 (8) ***	54 (7) ***	8 (3) *	-54 (7) ***	0.0024 (0.0003) ***	-0.0003 (0.0001) *	6 (1) **	9 (1) ***	-3 (1) **	-9 (1) ***	0.0133 (0.0015) ***	0.0049 (0.0010) ***
8	2X	76 (6) ***	70 (5) ***	6 (4)	-70 (5) ***	0.0031 (0.0002) ***	-0.0002 (0.0002)	6 (2) ***	10 (1) ***	-3 (1) ***	-10 (1) ***	0.0146 (0.0017) ***	0.0055 (0.0009) ***
4	5X	85 (9) ***	77 (9) ***	8 (4) *	-77 (9) ***	0.0034 (0.0004) ***	-0.0003 (0.0002)	8 (1) ***	13 (1) ***	-5 (1) ***	-13 (1) ***	0.0192 (0.0020) ***	0.0080 (0.0013) ***
8	5X	104 (8) ***	100 (7) ***	3 (5)	-100 (7) ***	0.0044 (0.0003) ***	-0.0001 (0.0002)	10 (2) ***	15 (1) ***	-6 (1) ***	-15 (1) ***	0.0229 (0.0018) ***	0.0092 (0.0011) ***

Supplementary Table 7. Average variant recall and precision rates in no-plexing WES and WEGS. The table represents variant calls inside the target regions in Agilent V7 capture and the GIAB high-confidence regions. The star symbols represent statistically significant differences between WES and WEGS when using a one-tailed Wilcoxon rank-sum test: * - P-value < 0.05, ** - P-value < 0.01, *** - P-value < 0.001. WEGS values in bold font are higher than the corresponding values in WES.

Sequencing method	Number of samples sequenced together (multiplexed)	WGS DP	SNVs		InDels	
			Recall (SE)	Precision (SE)	Recall (SE)	Precision (SE)
WES	1	–	0.9830 (0.0004)	0.9821 (0.0003)	0.9390 (0.0029)	0.9232 (0.0028)
$WEGS_{4P,2X}$	4	2	**0.9842 (0.0002)****	0.9812 (0.0002)*	**0.9493 (0.0028)***	**0.9269 (0.0024)**
$WEGS_{4P,5X}$	4	5	**0.9852 (0.0001)*****	0.9812 (0.0002)*	**0.9552 (0.0019)*****	**0.9300 (0.0024)***
$WEGS_{8P,2X}$	8	2	**0.9834 (0.0002)**	0.9814 (0.0003)	**0.9407 (0.0026)**	**0.9240 (0.0024)**
$WEGS_{8P,5X}$	8	5	**0.9847 (0.0001)*****	0.9816 (0.0002)	**0.9490 (0.0020)****	**0.9277 (0.0027)**

Supplementary Table 8. Average variant recall and precision rates in no-plexing WES and WEGS stratified by library preparation batch. The star symbols represent statistically significant differences between WES and WEGS in the same batch when using a one-tailed Wilcoxon rank-sum test: * - P-value < 0.05, ** - P-value < 0.01, *** - P-value < 0.001. WEGS values in bold font are higher than the corresponding values in WES in the same batch.

Batch	Label	Number of samples sequenced together (multiplexed)	WGS DP	SNVs		InDels	
				Recall (SE)	Precision (SE)	Recall (SE)	Precision (SE)
1	WES	1	0	0.9840 (0.0004)	0.9813 (0.0006)	0.9426 (0.0041)	0.9241 (0.0054)
1	$WEGS_{4P,2X}$	4	2	**0.9846 (0.0002)**	0.9812 (0.0003)	**0.9515 (0.0028)***	**0.9289 (0.0031)**
1	$WEGS_{4P,5X}$	4	5	**0.9854 (0.0002)****	0.9811 (0.0003)	**0.9569 (0.0020)****	**0.9315 (0.0032)**
1	$WEGS_{8P,2X}$	8	2	0.9838 (0.0003)	**0.9818 (0.0004)**	**0.9434 (0.0036)**	**0.9257 (0.0030)**
1	$WEGS_{8P,5X}$	8	5	**0.9849 (0.0002)***	**0.9819 (0.0004)**	**0.9506 (0.0029)**	**0.9293 (0.0040)**
2	WES	1	0	0.9826 (0.0004)	0.9824 (0.0003)	0.9371 (0.0038)	0.9228 (0.0036)
2	$WEGS_{4P,2X}$	4	2	**0.9835 (0.0002)**	0.9813 (0.0003)**	**0.9450 (0.0062)**	**0.9229 (0.0029)**
2	$WEGS_{4P,5X}$	4	5	**0.9849 (0.0002)****	0.9814 (0.0003)*	**0.9519 (0.0040)***	**0.9271 (0.0035)**
2	$WEGS_{8P,2X}$	8	2	**0.9830 (0.0002)**	0.9811 (0.0004)**	**0.9380 (0.0036)**	0.9223 (0.0038)
2	$WEGS_{8P,5X}$	8	5	**0.9845 (0.0002)****	0.9812 (0.0003)**	**0.9474 (0.0028)***	**0.9262 (0.0039)**

Supplementary Table 9. Average variant recall and precision rates in 30X WGS, 2X WGS, 5X WGS, WES, and WEGS. The table represents variant calls inside the target regions in Agilent V7 capture and the GIAB high-confidence regions.

Sequencing method	SNVs					InDels				
	TP (SE)	FP (SE)	FN (SE)	Precision (SE)	Recall (SE)	TP (SE)	FP (SE)	FN (SE)	Precision (SE)	Recall (SE)
30X WGS	22,338 (19)	260 (5)	287 (5)	0.9885 (0.0002)	0.9873 (0.0002)	661 (5)	12 (1)	11 (1)	0.9823 (0.0017)	0.9841 (0.0011)
2X WGS	12,387 (433)	3,158 (107)	10,238 (399)	0.7966 (0.0075)	0.5474 (0.0183)	359 (10)	257 (13)	313 (5)	0.5833 (0.0083)	0.5344 (0.0040)
5X WGS	17,347 (489)	1,857 (57)	5,278 (466)	0.9031 (0.0052)	0.7667 (0.0209)	494 (20)	280 (9)	177 (7)	0.6398 (0.0161)	0.7354 (0.0151)
WES	22,241 (30)	406 (7)	384 (8)	0.9821 (0.0003)	0.9830 (0.0004)	631 (6)	53 (2)	41 (2)	0.9232 (0.0028)	0.9390 (0.0029)
WEGS$_{4P,2X}$	22,268 (24)	426 (6)	357 (5)	0.9812 (0.0002)	0.9842 (0.0002)	638 (5)	51 (2)	34 (2)	0.9269 (0.0024)	0.9493 (0.0028)
WEGS$_{4P,5X}$	22,291 (25)	427 (6)	334 (3)	0.9812 (0.0002)	0.9852 (0.0001)	642 (5)	49 (2)	30 (1)	0.9300 (0.0024)	0.9552 (0.0019)
WEGS$_{8P,2X}$	22,247 (21)	421 (6)	376 (4)	0.9814 (0.0003)	0.9834 (0.0002)	633 (4)	52 (2)	40 (2)	0.9240 (0.0024)	0.9407 (0.0026)
WEGS$_{8P,5X}$	22,277 (21)	418 (6)	346 (3)	0.9816 (0.0002)	0.9847 (0.0001)	638 (4)	50 (2)	34 (2)	0.9277 (0.0027)	0.9490 (0.0020)

Supplementary Table 10. Average genome-wide variant recall and precision rates in 30X WGS, 2X WGS, 5X WGS, and WEGS. The table represents variant calls in genetic regions overlapping with the GIAB high-confidence regions genome-wide.

Sequencing method	SNV					InDel				
	TP (SE)	FP (SE)	FN (SE)	Precision (SE)	Recall (SE)	TP (SE)	FP (SE)	FN (SE)	Precision (SE)	Recall (SE)
30X WGS	3,309,667 (4,295)	15,268 (174)	26,097 (82)	0.9954 (0.0001)	0.9922 (0.0000)	500,728 (2,382)	5,605 (35)	9,757 (111)	0.9889 (0.0001)	0.9809 (0.0002)
2X WGS	1,190,984 (108,702)	322,983 (12,296)	2,144,781 (117,113)	0.7852 (0.0099)	0.3572 (0.0336)	115,029 (12,911)	52,321 (4,272)	395,456 (16,488)	0.6860 (0.0069)	0.2257 (0.0269
5X WGS	1,830,718 (144,541)	250,804 (7,048)	1,505,046 (153,117)	0.8780 (0.0113)	0.5490 (0.0449)	189,542 (19,319)	65,847 (3,181)	320,943 (22,545)	0.7404 (0.0103)	0.3718 (0.0404)
WEGS$_{4P,2X}$	1,333,840 (44,603)	303,452 (4,673)	2,001,925 (48,123)	0.8137 (0.0033)	0.4000 (0.0138)	132,918 (5,359)	56,441 (1,663)	377,567 (6,801)	0.7010 (0.0025)	0.2607 (0.0112)
WEGS$_{4P,5X}$	1,909,331 (58,921)	240,392 (3,054)	1,426,434 (62,566)	0.8869 (0.0043)	0.5726 (0.0183)	201,593 (7,908)	68,206 (1,221)	308,892 (9,249)	0.7457 (0.0041)	0.3954 (0.0166)
WEGS$_{8P,2X}$	1,326,086 (39,124)	307,039 (4,029)	2,008,327 (42,308)	0.8109 (0.0029)	0.3979 (0.0121)	131,783 (4,687)	56,193 (1,460)	378,097 (6,013)	0.7001 (0.0022)	0.2588 (0.0098)
WEGS$_{8P,5X}$	1,914,743 (51,710)	240,678 (2,681)	1,419,670 (54,999)	0.8870 (0.0038)	0.5745 (0.0161)	201,995 (6,927)	68,102 (1,067)	307,885 (8,174)	0.7463 (0.0036)	0.3967 (0.0146)

Supplementary Table 11. Precision and recall rates of variants imputed using the TOPMed reference panel inside WES target regions. P - precision. R - recall. For WEGS, this table reports average numbers for each sample. Each sample was sequenced 4 times using WEGS$_{4P,2X}$. HG002 and HG004 were sequenced 5 times using WEGS$_{8P,5X}$. HG003 was sequenced 6 times using WEGS$_{8P,5X}$. The percent of missed true variants is equal to (1 - recall) * 100.

Sample	TOPMed imputed					WEGS 4P, 2X					WEGS 8P, 5X				
	TP	FN	FP	P	R	TP (SE)	FN (SE)	FP (SE)	P (SE)	R (SE)	TP (SE)	FN (SE)	FP (SE)	P (SE)	R (SE)
							**SNVs**								
HG002	21,458	1,285	165	0.9924	0.9435	22,379 (7)	364 (7)	446 (6)	0.9805 (0.0002)	0.9840 (0.0003)	22,390 (5)	353 (5)	449 (7)	0.9803 (0.0003)	0.9845 (0.0002)
HG003	21,477	1,112	111	0.9949	0.9508	22,231 (9)	358 (9)	425 (4)	0.9813 (0.0001)	0.9841 (0.0004)	22,249 (6)	340 (6)	406 (5)	0.9821 (0.0002)	0.9850 (0.0003)
HG004	21,228	1,315	181	0.9915	0.9417	22,195 (9)	348 (9)	406 (7)	0.9820 (0.0003)	0.9846 (0.0004)	22,197 (5)	346 (5)	402 (3)	0.9822 (0.0001)	0.9847 (0.0002)
							**InDels**								
HG002	271	411	8	0.9713	0.3974	648 (3)	34 (3)	57 (1)	0.9197 (0.0017)	0.9498 (0.0050)	647 (2)	35 (2)	58 (3)	0.9177 (0.0038)	0.9481 (0.0028)
HG003	257	433	8	0.9698	0.3725	649 (2)	41 (2)	52 (2)	0.9254 (0.0019)	0.9409 (0.0027)	651 (1)	39 (1)	52 (1)	0.9265 (0.0018)	0.9432 (0.0019)
HG004	259	384	7	0.9738	0.4028	616 (2)	28 (2)	43 (2)	0.9357 (0.0035)	0.9572 (0.0026)	615 (2)	28 (2)	40 (2)	0.9393 (0.0033)	0.9568 (0.0031)

Supplementary Table 12. Precision and recall rates of variants imputed using the TOPMed reference panel genome-wide. P - precision. R - recall. For WEGS, this table reports average numbers for each sample. Each sample was sequenced 4 times using WEGS$_{4P,2X}$. HG002 and HG004 were sequenced 5 times using WEGS$_{8P,5X}$. HG003 was sequenced 6 times using WEGS$_{8P,5X}$. The percent of missed true variants is equal to (1 - recall) * 100.

Sample	TOPMed imputed			WEGS 4P,2X			WEGS 8P,5X		
	TP	P	R	TP (SE)	P (SE)	R (SE)	TP (SE)	P (SE)	R (SE)
				**SNVs**					
HG002	3,212,631	0.9934	0.9582	1,323,307 (13,949)	0.8183 (0.0023)	0.3947 (0.0042)	1,873,500 (1,770)	0.8853 (0.0002)	0.5588 (0.0005)
HG003	3,181,715	0.9947	0.96	1,518,733 (9,194)	0.8236 (0.0013)	0.4583 (0.0028)	2,151,757 (2,243)	0.9040 (0.0002)	0.6493 (0.0007)
HG004	3,202,593	0.9936	0.9587	1,159,478 (6,227)	0.7991 (0.0011)	0.3471 (0.0019)	1,671,569 (3,941)	0.8684 (0.0005)	0.5004 (0.0012)
				**InDels**					
HG002	199,347	0.9896	0.3816	132,493 (1,957)	0.7043 (0.0008)	0.2536 (0.0037)	198,767 (351)	0.7447 (0.0003)	0.3805 (0.0007)
HG003	195,885	0.9909	0.3911	154,695 (1,172)	0.7091 (0.0003)	0.3089 (0.0023)	232,882 (368)	0.7624 (0.0002)	0.4650 (0.0007)
HG004	197,298	0.9899	0.3882	111,566 (788)	0.6897 (0.0004)	0.2195 (0.0015)	168,158 (651)	0.7287 (0.0002)	0.3308 (0.0013)

Supplementary Table 13. Imputed variants, their allele frequencies, and overlap with true positive (TP) variants in WEGS outside WES target regions. The arrows ⇧ and ⇩ denote the increase and decrease in AF fold-change (AF ASJ / AF TOPMed) compared to variants where the number of imputed alleles matched the number of true alleles.

Sample	# of imputed alleles vs # of true alleles	N imputed variants	Median AF (Q1-Q3)			Mean % of variants which were TP in WEGS (SE)	
			ASJ	TOPMed	Fold-change	WEGS 4P,2X	WEGS 8P,5X
HG002	Same	3,258,732	0.460 (0.239-0.703)	0.442 (0.231-0.680)	1.042 (0.925-1.197)	38.44 (0.42)	55.16 (0.05)
HG002	Smaller	15,233	0.234 (0.059-0.483)	0.215 (0.043-0.453)	⇧ 1.136 (0.926-1.509)	41.85 (0.48)	59.31 (0.11)
HG002	Greater	17,161	0.329 (0.147-0.536)	0.340 (0.157-0.539)	⇩ 1.007 (0.840-1.209)	90.55 (0.05)	92.75 (0.02)
HG003	Same	3,225,810	0.461 (0.239-0.704)	0.443 (0.231-0.681)	1.043 (0.927-1.198)	44.84 (0.28)	64.45 (0.07)
HG003	Smaller	12,665	0.222 (0.051-0.496)	0.215 (0.033-0.458)	⇧1.129 (0.930-1.537)	46.75 (0.34)	67.09 (0.08)
HG003	Greater	13,855	0.302 (0.134-0.477)	0.318 (0.145-0.501)	⇩1.006 (0.822-1.210)	92.38 (0.04)	94.60 (0.03)
HG004	Same	3,247,624	0.457 (0.237-0.702)	0.440 (0.230-0.679)	1.041 (0.923-1.196)	33.65 (0.19)	49.22 (0.12)
HG004	Smaller	15,357	0.219 (0.056-0.465)	0.205 (0.042-0.440)	⇧1.114 (0.915-1.565)	38.89 (0.30)	55.00 (0.12)
HG004	Greater	16,457	0.325 (0.142-0.534)	0.330 (0.153-0.539)	⇩1.022 (0.841-1.239)	89.38 (0.05)	91.94 (0.02)

Supplementary Table 14. Precision and recall rates of GLIMPSE-imputed and called variants combined when using WEGS. P - precision. R - recall. This table reports the average number for each sample. Each sample was sequenced 4 times using $WEGS_{4P,2X}$. HG002 and HG004 were sequenced 5 times using $WEGS_{8P,5X}$. HG003 was sequenced 6 times using $WEGS_{8P,5X}$. The WES target regions are based on the Agilent V7 capture. Only variants inside the GIAB high-confidence regions were used for estimating precision and recall rates. The percent of missed true variants equals (1 - recall) * 100. The local imputation reference panel combines haplotypes from the 1000 Genomes Project and Human Genome Diversity Project (see Methods). The precision and recall rates for imputed variants when using low depth WGS data only are in Supplementary Tables 20 and 21.

	**GLIMPSE-imputed + called WEGS 4P,2X**					**GLIMPSE-imputed + called WEGS 8P,5X**				
**Sample**	**TP (SE)**	**FN (SE)**	**FP (SE)**	**P (SE)**	**R (SE)**	**TP (SE)**	**FN (SE)**	**FP (SE)**	**P (SE)**	**R (SE)**
					SNVs genome-wide					
HG002	2,453,912 (7,229)	898,768 (7,229)	135,769 (624)	0.9476 (0.0004)	0.7319 (0.0022)	2,766,979 (947)	585,700 (947)	121,292 (298)	0.9580 (0.0001)	0.8253 (0.0003)
HG003	2,671,670 (3,330)	642,473 (3,330)	149,397 (194)	0.9470 (0.0001)	0.8061 (0.0010)	2,941,974 (658)	372,168 (658)	111,323 (132)	0.9635 (0.0000)	0.8877 (0.0002)
HG004	2,288,272 (2,807)	1,052,199 (2,807)	134,772 (174)	0.9444 (0.0001)	0.6850 (0.0008)	2,627,490 (1,760)	712,981 (1,760)	124,847 (343)	0.9546 (0.0001)	0.7866 (0.0005)
					InDels genome-wide					
HG002	232,386 (1,580)	290,004 (1,580)	47,280 (661)	0.8310 (0.0010)	0.4449 (0.0030)	285,889 (275)	236,500 (275)	60,931 (123)	0.8243 (0.0002)	0.5473 (0.0005)
HG003	254,637 (814)	246,156 (814)	53,284 (451)	0.8270 (0.0008)	0.5085 (0.0016)	308,620 (243)	192,173 (243)	64,760 (153)	0.8266 (0.0002)	0.6163 (0.0005)
HG004	209,493 (584)	298,781 (584)	42,164 (395)	0.8325 (0.0009)	0.4122 (0.0011)	258,240 (489)	250,034 (489)	55,351 (264)	0.8235 (0.0004)	0.5081 (0.0010)
					SNVs inside target regions					
HG002	22,422 (1)	321 (1)	442 (7)	0.9807 (0.0003)	0.9859 (0.0001)	22,432 (5)	311 (5)	446 (8)	0.9805 (0.0003)	0.9863 (0.0002)
HG003	22,291 (3)	298 (3)	422 (5)	0.9814 (0.0002)	0.9868 (0.0001)	22,301 (4)	288 (4)	404 (4)	0.9822 (0.0002)	0.9872 (0.0002)
HG004	22,246 (6)	298 (6)	402 (6)	0.9823 (0.0003)	0.9868 (0.0003)	22,240 (3)	303 (3)	398 (3)	0.9824 (0.0001)	0.9865 (0.0001)
					InDels inside target regions					
HG002	649 (3)	33 (3)	58 (2)	0.9185 (0.0025)	0.9512 (0.0045)	647 (2)	35 (2)	59 (3)	0.9165 (0.0038)	0.9490 (0.0024)
HG003	649 (1)	41 (1)	54 (2)	0.9241 (0.0019)	0.9409 (0.0018)	652 (1)	38 (1)	52 (1)	0.9259 (0.0019)	0.9447 (0.0014)
HG004	618 (1)	25 (1)	42 (2)	0.9363 (0.0032)	0.9615 (0.0022)	619 (2)	24 (2)	40 (2)	0.9402 (0.0034)	0.9633 (0.0028)

Supplementary Table 15. Precision and recall rates of variants imputed using the GLIMPSE method and WEGS. P - precision. R - recall. This table reports the average number for each sample. Each sample was sequenced 4 times using WEGS4P,2X. HG002 and HG004 were sequenced 5 times using WEGS8P,5X. HG003 was sequenced 6 times using WEGS8P,5X. The WES target regions are based on the Agilent V7 capture. Only variants inside the GIAB high-confidence regions were used for estimating precision and recall rates. The percent of missed true variants equals (1 - recall) * 100. The local imputation reference panel combines haplotypes from the 1000 Genomes Project and Human Genome Diversity Project (see Methods). The precision and recall rates for imputed variants when using low depth WGS data only are in Supplementary Tables 20 and 21.

	**GLIMPSE-imputed WEGS 4P,2X**					**GLIMPSE-imputed WEGS 8P,5X**				
**Sample**	**TP (SE)**	**FN (SE)**	**FP (SE)**	**P (SE)**	**R (SE)**	**TP (SE)**	**FN (SE)**	**FP (SE)**	**P (SE)**	**R (SE)**
SNVs genome-wide										
HG002	2,374,106 (5,976)	978,573 (5,976)	14,526 (105)	0.9939 (0.0001)	0.7081 (0.0018)	2,650,201 (769)	702,478 (769)	11,347 (20)	0.9957 (0.0000)	0.7905 (0.0002)
HG003	2,585,159 (2,514)	728,984 (2,514)	14,954 (78)	0.9942 (0.0000)	0.7800 (0.0008)	2,814,345 (432)	499,798 (432)	10,790 (23)	0.9962 (0.0000)	0.8492 (0.0001)
HG004	2,220,297 (2,243)	1,120,174 (2,243)	15,322 (56)	0.9931 (0.0000)	0.6647 (0.0007)	2,526,333 (1,400)	814,138 (1,400)	12,130 (55)	0.9952 (0.0000)	0.7563 (0.0004)
InDels genome-wide										
HG002	173,796 (456)	348,592 (456)	1,960 (20)	0.9889 (0.0001)	0.3327 (0.0009)	192,324 (58)	330,065 (58)	1,671 (8)	0.9914 (0.0000)	0.3682 (0.0001)
HG003	186,398 (148)	314,395 (148)	1,923 (14)	0.9898 (0.0001)	0.3722 (0.0003)	200,140 (35)	300,653 (35)	1,611 (5)	0.9920 (0.0000)	0.3996 (0.0001)
HG004	161,474 (133)	346,800 (133)	1,878 (4)	0.9885 (0.0000)	0.3177 (0.0003)	181,722 (101)	326,552 (101)	1,725 (7)	0.9906 (0.0000)	0.3575 (0.0002)
SNVs inside WES targeted regions										
HG002	20,875 (1)	1,868 (1)	30 (1)	0.9986 (0.0000)	0.9179 (0.0000)	20,878 (1)	1,865 (1)	31 (2)	0.9985 (0.0001)	0.9180 (0.0001)
HG003	20,869 (1)	1,720 (1)	29 (1)	0.9986 (0.0000)	0.9239 (0.0000)	20,871 (1)	1,718 (1)	29 (1)	0.9986 (0.0000)	0.9240 (0.0000)
HG004	20,746 (2)	1,797 (2)	28 (1)	0.9987 (0.0000)	0.9203 (0.0001)	20,746 (1)	1,797 (1)	29 (1)	0.9986 (0.0000)	0.9203 (0.0000)
InDels inside WES targeted regions										
HG002	308 (0)	374 (0)	4 (1)	0.9856 (0.0021)	0.4509 (0.0004)	307 (0)	375 (0)	4 (0)	0.9859 (0.0013)	0.4504 (0.0003)
HG003	292 (0)	398 (0)	2 (0)	0.9932 (0.0000)	0.4232 (0.0000)	293 (0)	397 (0)	2 (0)	0.9932 (0.0000)	0.4242 (0.0003)
HG004	306 (0)	337 (0)	2 (0)	0.9935 (0.0000)	0.4755 (0.0004)	306 (0)	337 (0)	2 (0)	0.9922 (0.0008)	0.4759 (0.0007)

Supplementary Table 16. Precision and recall rates of variants imputed using the Minimac4 method genome-wide. The percent of missed true variants equals (1 - recall) * 100. The local imputation reference panel combines haplotypes from the 1000 Genomes Project and Human Genome Diversity Project (see Methods).

	**Minimac4-imputed SNVs**					**Minimac4-imputed InDels**				
**Sample**	**TP**	**FN**	**FP**	**Precision**	**Recall**	**TP**	**FN**	**FP**	**Precision**	**Recall**
HG002	2,942,195	410,484	316,718	0.9028	0.8776	326,360	196,029	47,144	0.8738	0.6247
HG003	2,908,634	405,509	317,430	0.9016	0.8776	317,001	183,792	46,494	0.8721	0.633
HG004	2,934,901	405,570	313,069	0.9036	0.8786	320,526	187,748	45,098	0.8767	0.6306

Supplementary Table 17. **Overview of genome-wide significant loci associated with peripheral artery disease (PAD) in the 862 WEGS sequenced patients**. Abbreviations: chr-chromosome; alt-alternative; ref-reference; freq-frequency; EUR-Europeans; AA-African Americans; EAS-East Asians; DP-average depth; GSA-global screening array (24v3). This table reports the allele frequency and average depth of known genome-wide significant peripheral artery disease loci in the WEGS. The ancestry specific frequency was derived from gnomAD (v3.1.2).

**rsid**	**chr**	**position**	**Alt**	**Ref**	**Alt Freq**	**EUR Freq**	**AA Freq**	**EAS Freq**	**MID Freq**	**DP**	**Annotation**	**Gene /Locus**	**Present on the GSA array**
rs7528419	1	109274570	G	A	0.16	0.22	0.27	0.06	0.10	7	3' UTR variant	*CELSR2/SORT1*	+
rs6025	1	169549811	T	C	0.05	0.03	0.004	0.000	0.06	44	Missense variant	*F5*	+
rs118039278	6	160564494	A	G	0.08	0.07	0.01	0.001	0.01	7	Intron variant	*LPA*	-
rs3130968	6	31097294	T	C	0.11	0.14	0.15	0.001	0.05	9	Regulatory region variant	*HLA-B*	-
rs2107595	7	19009765	A	G	0.14	0.16	0.21	0.34	0.22	5	Regulatory region variant	*HDAC9*	-
rs4722172	7	22746913	A	G	0.74	0.79	0.96	1.000	0.91	7	Intergenic variant	*IL6*	-
rs322	8	19961706	C	A	0.22	0.27	0.40	0.20	0.28	4	Intron variant	*LPL*	-
rs505922	9	133273813	T	C	0.56	0.65	0.65	0.56	0.66	7	Intron variant	*ABO*	+
rs1537372	9	22103184	T	G	0.35	0.48	0.14	0.52	0.63	6	Intron variant	*CDKN2B-AS1/*9p21	
rs7903146	10	112998590	T	C	0.17	0.29	0.29	0.03	0.40	3	Intron variant	*TCF7L2*	+
rs7476	11	46321284	C	A	0.22	0.21	0.84	0.46	0.41	4	Intron variant	*MMP3*	-
rs566125	11	102839740	T	C	0.12	0.13	0.02	0.07	0.13	5	3' UTR variant	*CREB3L1*	-
rs4842266	12	79557786	A	G	0.50	0.44	0.07	0.002	0.37	4	Intron variant	*PTPN11*	+
rs11066301	12	112433568	G	A	0.34	0.44	0.07	0.002	0.37	111	Upstream gene variant	*RP11-359M6.3*	-
rs1975514	13	110176544	C	T	0.37	0.37	0.28	0.30	0.31	8	Intron variant	*COL4A1*	-
rs55784307	14	70034647	A	C	0.15	0.18	0.05	0.39	0.13	5	Downstream gene variant	*SMOC1*	+
rs10851907	15	78623522	A	G	0.34	0.42	0.43	0.07	0.44	5	Upstream gene variant	*CHRNA3*	-
rs138294113	19	11081053	T	C	0.09	0.12	0.14	0.01	0.16	6	Intergenic variant	LDLR	-

Supplementary Table 18. Genome-wide significant loci associated with peripheral artery disease (PAD) and the number of variants within the loci present in WEGS and TOPMed. For each locus, we counted the number of variants surrounding the lead variant (rsid) within ±500 kilobase (kb) distance.

rsid	chr	position	# of SNVs in WEGS	# of SNVs in TOPMed	# of WEGS SNVs absent from TOPMed					
					Total	Synon	Non-synon	Stop/Splice	Frameshift	Inframe
rs7528419	1	109274570	17,631	258,859	4,481	17	35	2	2	0
rs6025	1	169549811	14,663	254,419	3,310	9	8	3	0	0
rs118039278	6	160564494	15,028	272,156	3,141	1	17	0	0	0
rs3130968	6	31097294	32,930	255,957	6,085	24	64	5	10	10
rs2107595	7	19009765	14,424	322,455	2,379	4	2	0	0	0
rs4722172	7	22746913	15,796	276,263	3,505	3	7	1	3	0
rs322	8	19961706	17333	333,151	3,251	4	10	1	0	0
rs505922	9	133273813	14,233	290,358	4,274	9	29	1	1	3
rs1537372	9	22103184	20,034	324,269	2,731	3	2	0	0	0
rs7903146	10	112998590	14,599	268,399	3,304	1	3	0	0	0
rs566125	11	46321284	16,485	262,361	4,932	8	34	1	0	0
rs7476	11	102839740	15,023	274,797	3,198	4	23	0	1	0
rs11066301	12	79557786	55,094	262,291	3,143	1	3	0	0	0
rs4842266	12	112433568	16,233	250,632	5,016	3	9	2	0	0
rs1975514	13	110176544	17,779	288,888	3,274	7	25	1	0	2
rs55784307	14	70034647	15,766	264,425	3,387	4	12	0	0	0
rs10851907	15	78623522	18,510	275,484	4,514	7	25	1	0	2
rs62084752	17	68093252	21,373	285,583	5,903	7	11	0	2	0
rs138294113	19	11081053	25,994	306,166	7,227	21	34	3	4	2

Supplementary Table 19. Precision and recall rates of variants imputed from 2X and 5X WGS using the GLIMPSE method. P - precision. R – recall. Each sample was sequenced one time using 5X WGS with reads split into two lanes. The WES target regions are based on the Agilent V7 capture. Only variants inside the GIAB high-confidence regions were used for estimating precision and recall rates. The percent of missed true variants equals (1 - recall) * 100. The local imputation reference panel combines haplotypes from the 1000 Genomes Project and Human Genome Diversity Project (see Methods).

Sample	GLIMPSE-imputed 2X WGS					GLIMPSE-imputed 5X WGS				
	TP	FN	FP	P	R	TP	FN	FP	P	R
SNVs genome-wide										
HG002	2,307,826	1,044,853	15,652	0.9933	0.6884	2,623,366	729,313	11,810	0.9955	0.7825
HG003	2,542,792	771,351	15,847	0.9938	0.7673	2,801,288	512,855	11,235	0.9960	0.8453
HG004	2,141,392	1,199,079	16,572	0.9923	0.6410	2,493,060	847,411	12,636	0.9950	0.7463
InDels genome-wide										
HG002	168,584	353,805	1,995	0.9883	0.3227	190,138	332,251	1,753	0.9909	0.364
HG003	183,474	317,319	2,039	0.9890	0.3664	199,136	301,657	1,670	0.9917	0.3976
HG004	155,368	352,906	1,941	0.9877	0.3057	179,054	329,220	1,779	0.9902	0.3523
SNVs inside WES target regions										
HG002	18,993	3,750	130	0.9932	0.8351	19,966	2,777	86	0.9957	0.8779
HG003	19,594	2,995	157	0.9921	0.8674	20,467	2,122	85	0.9959	0.9061
HG004	18,360	4,183	152	0.9918	0.8144	19,509	3,034	91	0.9954	0.8654
InDels inside WES target regions										
HG002	285	397	13	0.9564	0.4179	296	386	12	0.9610	0.434
HG003	275	415	9	0.9683	0.3986	285	405	9	0.9694	0.413
HG004	275	368	11	0.9617	0.4277	288	355	10	0.9666	0.4479

Supplementary Table 20. Precision and recall rates of GLIMPSE-imputed and called variants combined when using 2X and 5X WGS. P - precision. R – recall. Each sample was sequenced one time using 5X WGS with reads split into two lanes. The WES target regions are based on the Agilent V7 capture. Only variants inside the GIAB high-confidence regions were used for estimating precision and recall rates. The percent of missed true variants equals (1 - recall) * 100. The local imputation reference panel combines haplotypes from the 1000 Genomes Project and Human Genome Diversity Project (see Methods).

	GLIMPSE-imputed + called 2X WGS					GLIMPSE-imputed + called 5X WGS				
**Sample**	**TP**	**FN**	**FP**	**P**	**R**	**TP**	**FN**	**FP**	**P**	**R**
SNVs genome-wide										
HG002	2,375,840	976,839	144,901	0.9425	0.7086	2,734,168	618,511	125,112	0.9562	0.8155
HG003	2,618,513	695,630	158,730	0.9428	0.7901	2,923,651	390,492	114,796	0.9622	0.8822
HG004	2,197,878	1,142,593	142,826	0.939	0.6580	2,588,113	752,358	128,663	0.9526	0.7748
InDels genome-wide										
HG002	217,227	305,162	42,767	0.8355	0.4158	277,580	244,809	58,603	0.8257	0.5314
HG003	243,239	257,554	49,387	0.8313	0.4857	302,734	198,059	63,071	0.8276	0.6045
HG004	194,140	314,134	37,167	0.8393	0.3820	249,665	258,609	52,795	0.8255	0.4912
SNVs inside WES target regions										
HG002	19,763	2,980	1,861	0.9139	0.8690	21,116	1,627	1,315	0.9414	0.9285
HG003	20,342	2,247	2,092	0.9067	0.9005	21,517	1,072	1,266	0.9444	0.9525
HG004	19,069	3,474	1,943	0.9075	0.8459	20,536	2,007	1,324	0.9394	0.911
InDels inside WES target regions										
HG002	490	192	274	0.6423	0.7185	571	111	276	0.6749	0.8372
HG003	483	207	247	0.6616	0.7000	574	116	267	0.6833	0.8319
HG004	457	186	231	0.6647	0.7107	527	116	295	0.6442	0.8196
